# Phthalide mono- and dimers from the rhizomes of *Angelica sinensis* and their anti-inflammatory activities

**DOI:** 10.1007/s13659-025-00512-z

**Published:** 2025-04-24

**Authors:** Hongyan Wen, Sheng Li, Yu Zhang

**Affiliations:** 1 College of Ethnic Medicine, Yunnan University of Chinese Medicine, Kunming, 650500 China; 2 State Key Laboratory of Phytochemistry and Natural Medicines, Kunming Institute of Botany, Chinese Academy of Sciences, Kunming, 650201 China

**Keywords:** *Angelica sinensis*, Phthalide dimers, Angesicolides A and B, Anti-inflammatory activity

## Abstract

Three pairs of enantiomeric phthalide dimers, including two new ones, angesicolides A (**1**) and B (**2**), and a new phthalide monomer (**3**), were obtained from the rhizomes of *Angelica sinensis*. Their structures were established through spectroscopic methods, quantum calculations, and chiral HPLC analysis. Compounds **1** and **2** were [2 + 2] and [4 + 2] cycloadducts of phthalide monomers, and their hypothetical biogenetic origin was proposed. Compounds **2**, (+)-**2**, (−)-**2**, **4**, (+)-**4**, and (−)-**4** exhibited significant inhibitory activity against NO production with IC_50_ values range from 1.23 to 5.55 μM.

**Graphical Abstract:**

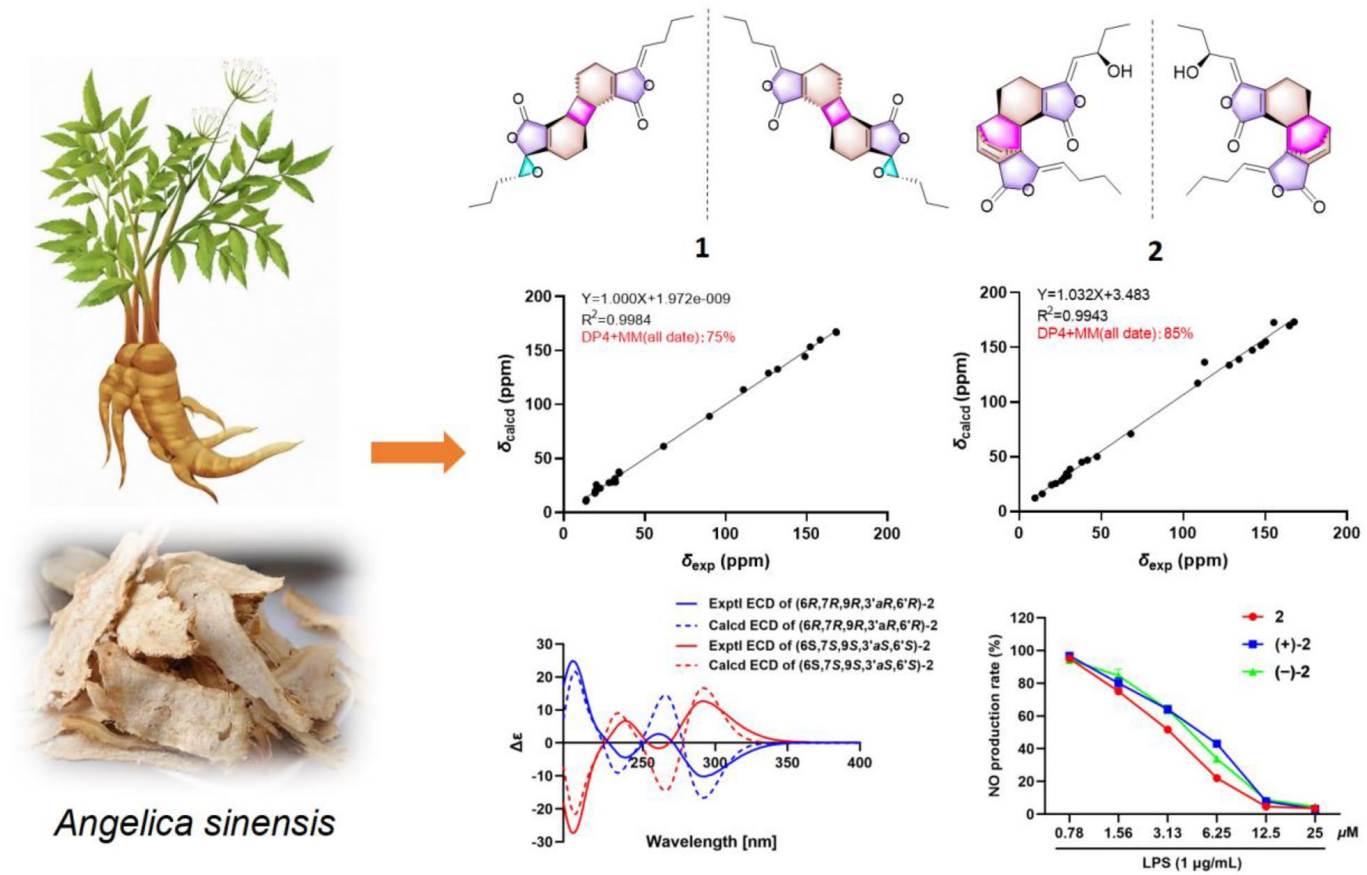

**Supplementary Information:**

The online version contains supplementary material available at 10.1007/s13659-025-00512-z.

## Introduction

The roots of *Angelica sinensis* (Oliv.) Diels (Umbelliferae family) is a well-known food and medicine homology herb, which has been widely used as therapeutic treatment for menstrual disorders and chronic constipation for thousands of years [1, 2]. Till now, there are more than ten types of secondary metabolisms have been identified from *A. sinensis* [3]. Among them, ligustilide and other structure intriguing dimeric phthalides were the representative components [4]. Their diverse structures and biological activities including anti-inflammatory, anticonvulsion, and platelet aggregation inhibition are long-standing attractive targets among natural product chemists and pharmacologists [5–8].

**Fig. 1:**
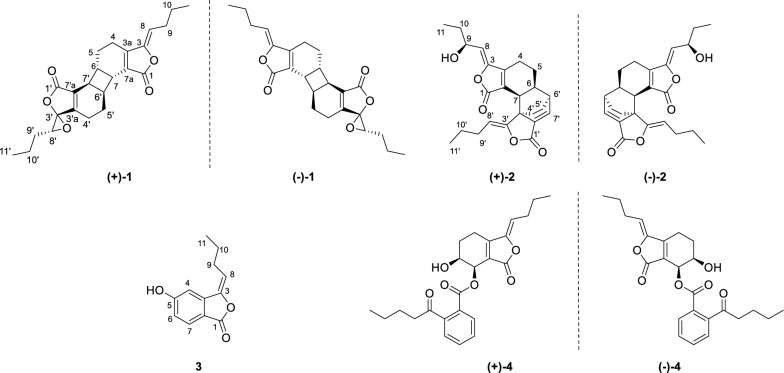
Chemical structures of compounds **1**–**4**

NO is synthesized and released into endothelial cells by nitric oxide synthase and plays vital role in the pathogenesis of inflammatory diseases [9]. Moreover, excessive production of NO is closely related to a variety of inflammatory disorders [10, 11]. Some phthalide dimers have been found to have significant anti-inflammation activity by inhibiting LPS-induced NO production [12]. Recently, an increasing number of studies have been reported on natural products with remarkable biological activities and novel structures from medicinal and dietary plants [13, 14]. To further explore anti-inflammatory natural products from medicinal plants, four phthalides including three pairs of enantiomeric dimers and a monomer (Fig. 1) were isolated and identified from the rhizomes of *A. sinensis*. Herein, we describe their structural elucidation and anti-inflammatory activities.

## Results and discussion

**Fig. 2:**
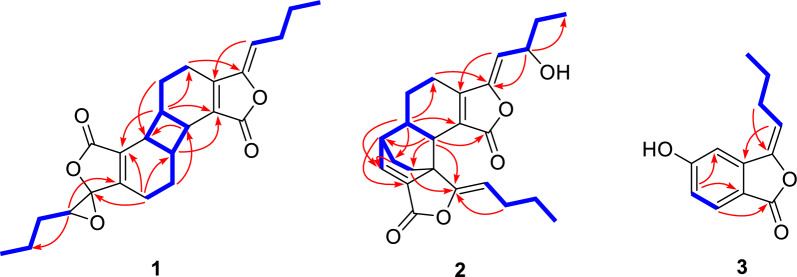
Key ^1^H–.^1^H COSY (bold lines) and HMBC (arrows) correlations of compounds **1**–**3**

Angesicolide A (**1**) was isolated as colorless oil. The HR-ESI–MS of **1** (*m*/*z* 397.2015 [M + H]^+^, calcd for 397.2010) indicated its molecular formula of C_24_H_28_O_5_, with 11 degrees of unsaturation. IR band at 1758 cm^−1^ indicated the presence of an α,β-unsaturated lactone. Compound **1** demonstrated 24 carbon signals in ^13^C NMR, which was classified into two carbonyl carbons (*δ*_C_ 168.4, 168.2), six olefinic carbons, two oxygenated carbons (*δ*_C_ 89.9, 61.6), four methines, eight methylenes, two methyls (*δ*_C_ 13.8, 13.6) by DEPT experiments. The ^1^H NMR (Table 1) demonstrated the presence of two butylidene side chains [*δ*_H_ 5.11 (1H), 2.33 (2H), 1.47 (2H), and 0.93 (3H); *δ*_H_ 3.27 (1H), 1.90, 1.78 (each 1H), 1.54 (2H), and 0.93 (3H)], four methine signals (*δ*_H_ 3.04, 3.58, 3.03, 3.57) and four methane signals [*δ*_H_ 2.35 (2H), 1.80 (2H), 2.17, 2.04 (each 1H), 1.86 (2H)]. These signals suggested **1** to be a phthalide dimer with the same basic skeleton as chaxiongnolide A [15]. Striking differences included an extra epoxy group based on same degree of unsaturation and the absence of a double bond. The crucial HMBC correlation between H-8ʹ (*δ*_H_ 3.27) with C-10ʹ (*δ*_C_ 19.2), C-9ʹ (*δ*_C_ 29.8), and C-3ʹa (*δ*_C_ 158.4) indicated that the epoxy group was situated at C-3ʹ and C-8ʹ. Further ^1^H–^1^H COSY, HSQC, and HMBC data verified the constructive planar structure (Fig. 2).

**Table 1:** NMR (500 MHz) Data of **1**–**3** (*δ* in ppm, *J* in Hz)

No	1^a^		2^a^		3^b^	
	*δ*_C_	*δ*_H_	*δ*_C_	*δ*_H_	*δ*_C_	*δ*_H_
1	168.4		167.7		168.8	
3	148.9		147.5		147.1	
3a	152.3		155.3		142.0	
4a	19.4	2.35, m^c^	19.7	2.20, m	109.9	7.23, d (2.0)
4b		2.35, m^c^		2.08, m		
5a	20.1	1.80, m^c^	28.8	1.95, m	165.3	
5b		1.80, m^c^		1.55, m		
6	34.3	3.04, m	38.2	2.56, t (7.5)	119.3	7.00, dd (8.5, 2.0)
7	31.9	3.58, d (7.0)	41.6	3.25, d (9.0)	127.9	7.70, d (8.5)
7a	126.4		128.0		118.1	
8	110.9	5.11, t (8.0)	113.0	5.08, d (8.5)	114.7	5.81, t (8.0)
9a	27.9	2.33, m^c^	68.1	4.64, m	28.9	2.50, q (7.5)^c^
9b		2.33, m^c^				2.50, q (7.5)^c^
10a	22.5	1.47, q (7.0)^c^	29.9	1.66, m^c^	23.9	1.62, m^c^
10b		1.47, q (7.0)^c^		1.66, m^c^		1.62, m^c^
11	13.8	0.93, m	9.6	0.92, t (5.0)	14.1	1.04, t (5.0)
1′	168.2		164.8			
3′	89.9		150.3			
3′a	158.4		47.5			
4′a	19.5	2.17, m	31.0	2.04, m		
4′b		2.04, m		1.42, m		
5′a	20.0	1.86, m^c^	25.8	1.89, m		
5′b		1.86, m^c^		1.30, m		
6′	34.1	3.03, m	41.5	2.99, m		
7′	31.6	3.57, d (7.0)	142.2	7.35, d (6.5)		
7′a	132.1		134.1			
8′	61.6	3.27, t (6.5)	108.8	4.99, t (7.5)		
9′a	29.8	1.90, m	27.5	2.16, m^c^		
9′b		1.78, m		2.16, m^c^		
10′a	19.2	1.54, m^c^	22.3	1.45, m^c^		
10′b		1.54, m^c^		1.45, m^c^		
11′	13.6	0.93, m	14.0	0.93, t (5.5)		

^a^Measured in CDCl_3_

^b^Measured in methanol-*d*_4_

^c^Overlapped

**Fig. 3:**
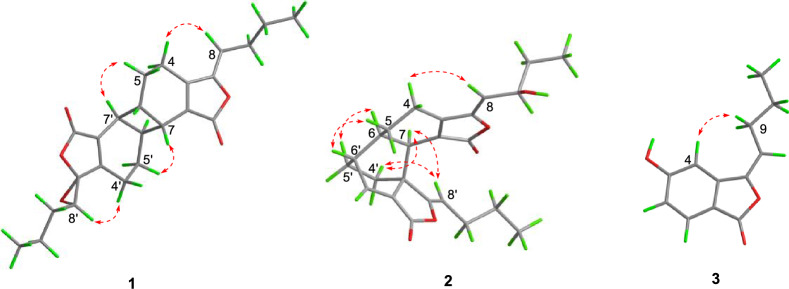
Key ROESY (dashed lines) correlations of **1**–**3**

**Fig. 4:**
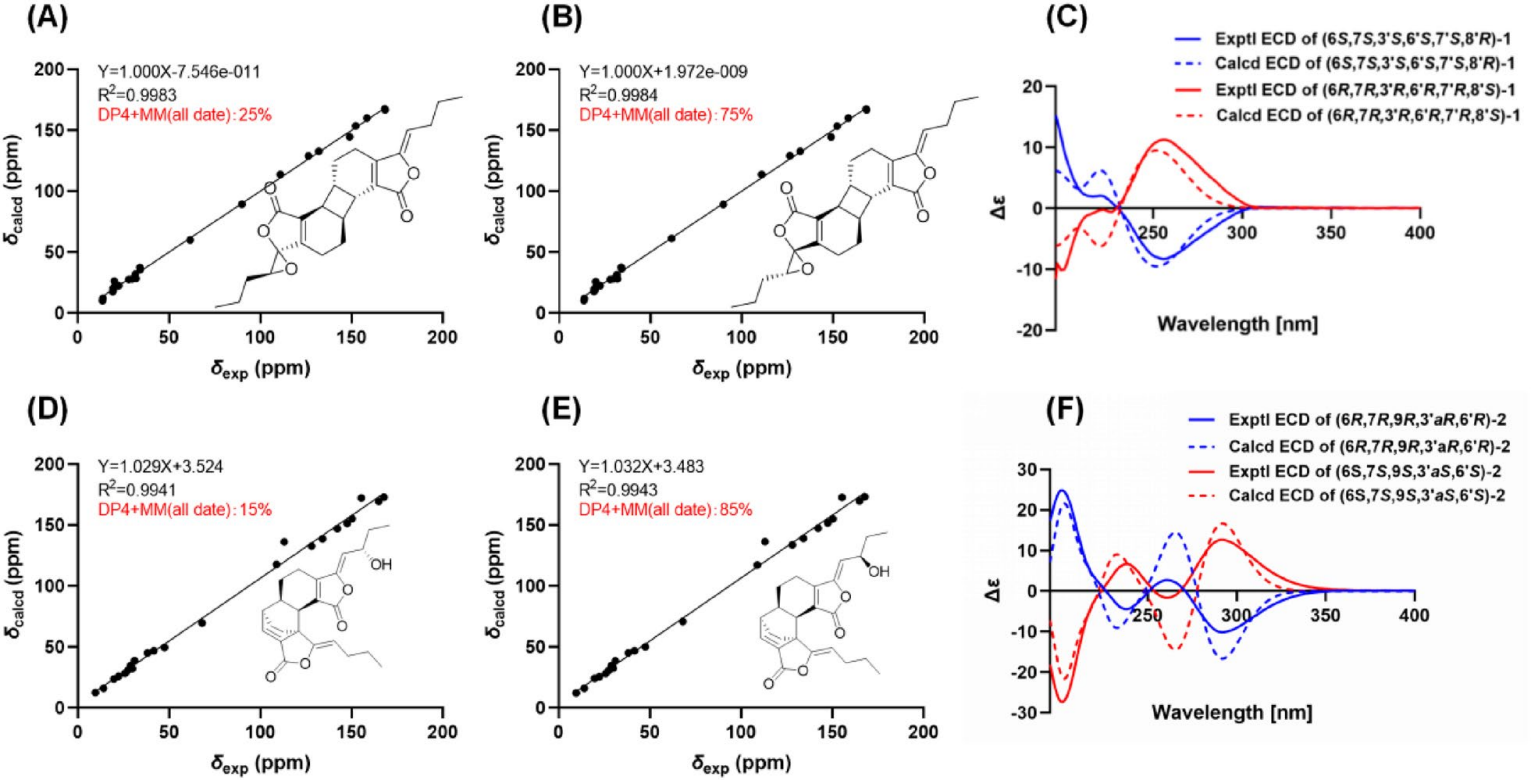
The.^13^C NMR calculation results of two plausible stereoisomers and DP4 + MM probability analysis at wB97XD/6–31+G(d,p) level. **A**,**B** Linear correlation plots of calculated vs. experimental NMR chemical shift values and DP4 + MM probability analysis for **1A** and **1B**; **C** comparison of the experimental and calculated ECD spectra of **1**. **D**,**E** Linear correlation plots of calculated vs. experimental NMR chemical shift values and DP4 + MM probability analysis for **2A** and **2B**; **F** comparison of the experimental and calculated ECD spectra of **2**

The relative stereochemistry of **1** was elucidated based on interpretation of ROESY interactions. The butylidene side chain took *Z*-form by the ROESY correlation of H-8 and H-4a. Furthermore, the ROESY correlations of H-7′/H-5a and H-7/H-5′a indicated *β*-orientations for H-6 and H-7, and *α*-orientations for H-6′ and H-7′. Consequently, compound **1** was determined to possess the relative configurations of 6R*,7R*,6′R*, and 7′R* (Fig. 3). Although H-4′a showed a strong ROESY correlation with H-8′, it was insufficient to determine the relative configuration of C-3′ and C-8′. Therefore, two possible stereoisomers have been proposed: (6R*,7R*,3′R*,6′R*,7′R*,8′S*)-**1A** or (6R*,7R*,3′S*,6′R*,7′R*,8′R*)-**1B**. These configurations were subsequently subjected to NMR calculations by employing the GIAO approach at the wB97XD/6–31+G(d,p) level of theory [16]. The results indicated the whole relative configuration of **1** to be 6R*,7R*,3′R*,6′R*,7′R*,8′S* with a DP4 + MM probability of approximately 75% (Fig. 4B).

Angesicolide B (**2**) was obtained as colorless gum. **2** had the molecular formula of C_24_H_28_O_5_ deduced by the HR-ESI–MS data (*m*/*z* 414.2271 [M + NH_4_]^+^, calcd for C_24_H_28_O_5_NH_4_ 414.2275), with 11 degrees of unsaturation. The existence of hydroxy and *α*,*β*-unsaturated lactone moieties was evidenced by the IR absorption bands at 3437, 1772 and 1710 cm^−1^. **2** showed 24 carbon signals including eight quaternary carbons, seven methines, seven methylenes and two methyls. The high similarity NMR data with that of levulanolide A indicated both compounds shared the same carbon skeleton [17], except for the existence of an additional hydroxyl group in **2** (*δ*_H_ 4.64; *δ*_C_ 68.1). The HMBC correlations of H-9 (*δ*_H_ 4.64) with C-8 (*δ*_C_ 113.0) and C-10 (*δ*_C_ 29.9) finally established the hydroxyl group was attached to C-9 (Fig. 2).

The *α*-orientations of H-6 and H-7, and the *β*-orientation of H-6′ were confirmed by the ROESY correlations of H-4′a/H-7, H-4′a/H-8′, H-7/H-8′, H-5/H-6′, and H-6/H-5′. Consequently, the relative configurations of C-6, C-7, C-3′a, and C-6′ were assigned as 6R*,7R*,3′aR*, and 6′R*, respectively (Fig. 3). The stereochemistry of the hydroxyl group at C-9 could not be determined through ROESY correlations. Consequently, two possible relative configurations were proposed for compound **2**: (6R*,7R*,9R*,3′aR*,6′R*)-**2A** and (6R*,7R*,9S*,3′aR*,6′R*)-**2B**. The subsequent experimental and the calculated NMR data established the relative configuration of **2** as 6R*,7R*,9R*,3′aR*,6′R*, with a probability of approximately 85% for DP4 + MM (Fig. 4E).

Angesicolide C (**3**) was purified as yellow oil. It had the same molecular formula of C_12_H_12_O_3_ with that of senkyunolide C [18], based on the HR-ESI–MS data at *m*/*z* 205.0861 [M + H]^+^ (calcd for 205.0859). Upon analysis of the 1D and 2D NMR data, **3** possessed the same planar structure as that of senkyunolide C. The most pronounced difference was the enolicdouble bond in **3** took *E*-configuration, as verified by the absence of ROESY correlation of H-8 and H-4 and the corresponding upfield chemical shifts at C-4 (Δ*δ*_C_ − 8.2 ppm) and C-8 (Δ*δ*_C_ − 3.7 ppm) in **3**.

Based on the specific optical rotation and chiral HPLC data, compounds **1** and **2** were a mixture of enantiomers, which were further treated by chiral separation to give two pairs of enantiomers [(+)-**1**/(−)-**1**, (+)-**2**/(−)-**2**]. As shown in Fig. 4, the calculated ECD results finally established the absolute configurations of (+)-**1** and (−)-**1** as (6*S*,7*R*,3ʹ*S*,6′*S*,7′*R,*8ʹ*R*)-**1** and (6*R*,7*S*,3ʹ*R*,6′*R*,7′*S,*8ʹ*S*)-**1** (Fig. 4C), respectively. Similarly, the absolute configurations of (+)-**2** and (−)-**2** were assigned as (6*R*,7*R*,9*R*,3′a*R*,6′*R*)-**2** and (6*S*,7*S*,9*S*,3′a*S*,6′*S*)-**2** (Fig. 4F).

Compound **4** was firstly isolated from *A. sinensis*. Comparing its spectral data with the reported data, compound **4** was identified as (±)-lyocasuarolide A [19]. Compounds (+)-**4** and (−)-**4** were obtained by chiral HPLC analysis, see Supporting Information for details.

**Fig. 5:**
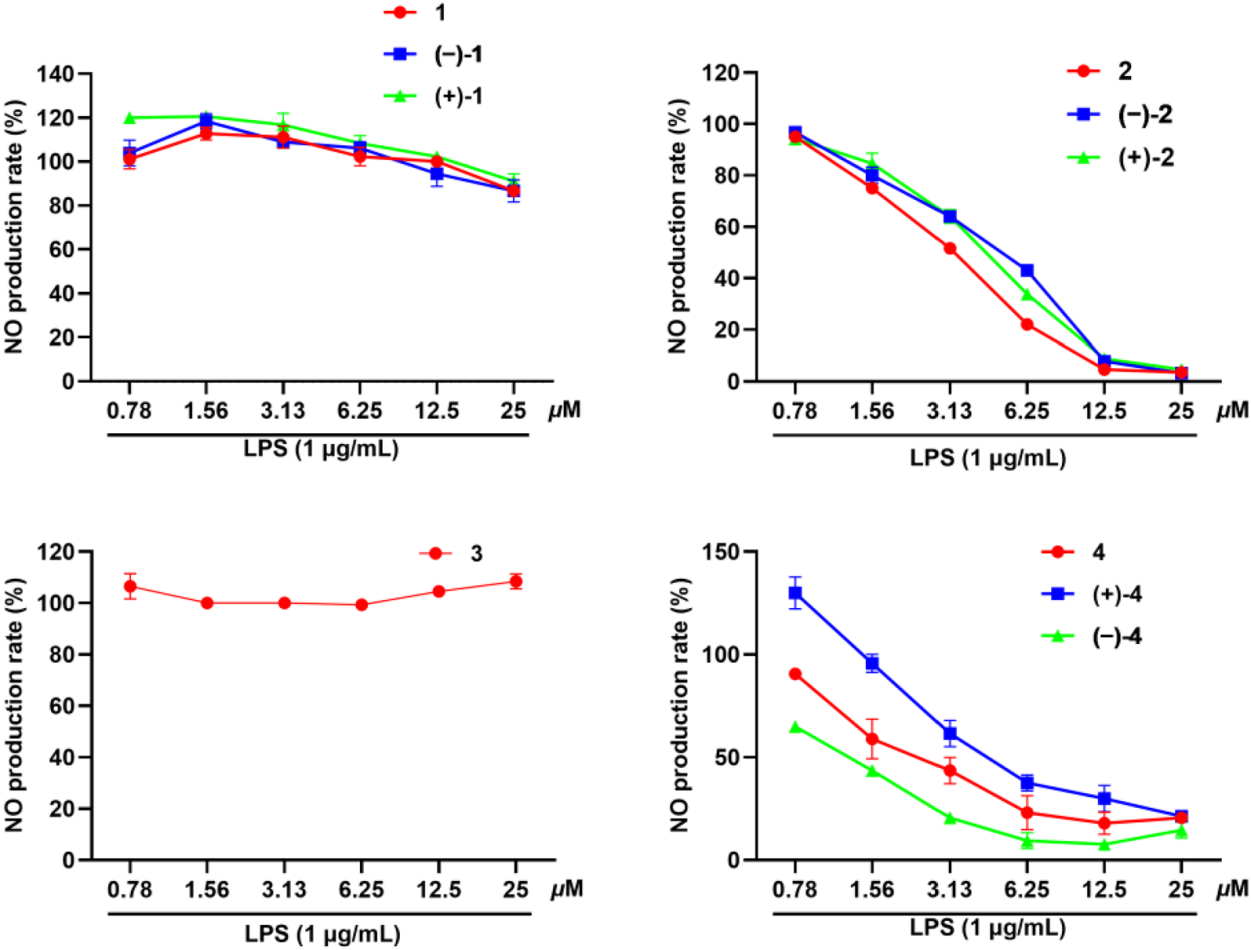
The inhibition rates of compounds **1**–**4** against LPS-induced NO production in RAW 264.7 cells

All the isolates (**1**–**4**) were evaluated in vitro for their inhibitory effects against NO production in LPS-induced RAW 246.7 mouse macrophages. As shown in Fig. 5 and Figure S1, compounds **2** and **4** and their enantiomers exhibit inhibitory activity against LPS-induced NO production without cytotoxicity. As shown in Table 2, compounds **2**, (+)-**2**, (−)-**2**, **4**, (+)-**4**, and (−)-**4** exhibited significant NO inhibitory effects with IC_50_ values between 1.23 and 5.55 μM, which were more potential than the positive control l-NMMA.

**Table 2:** IC_50_ values of compounds **1**–**4** and l-NMMA against LPS-induced NO production in RAW 264.7 cells

Compounds	Inhibition of NO production	
	IC_50_ (μM)	NO inhibition at 25 μM (%)
**1**	ND^a^	13.33 ± 2.54
(+)-**1**	ND^a^	8.89 ± 3.33
(−)-**1**	ND^a^	13.33 ± 5.09
**2**	3.12	96.44 ± 2.22
(+)-**2**	4.20	95.37 ± 1.63
(−)-**2**	4.44	96.80 ± 1.63
**3**	ND^a^	–
**4**	2.70	79.49 ± 2.96
(+)-**4**	5.55	78.63 ± 2.56
(−)-**4**	1.23	85.47 ± 3.92
l-NMMA^b^	33.0	38.32 ± 4.89

^a^ND, not detected

^b^l-NMMA, positive control

## Materials and methods

### General experimental procedures

NMR, HR-ESI–MS, UV, and IR spectra were measured as described previously [20].

### Plant materials

Details regarding the plant material, including collection and identification information, have been reported previously [20].

### Extraction and isolation

Detailed information for compounds **1**–**4** isolation procedures please see the Supporting Information.

### Chiral separation and characterization

Compounds **1** and **2** were analyzed and isolated with a CHIRALPAK AD-H chiral column to give (+)-**1**/(−)-**1**, (+)-**2**/(−)-**2**. Chiral separation details are shown in the Supporting Information.

Compound **1**: Colorless oil; UV (MeOH) *λ*_max_ (log *ε*) 278 (3.56) nm; HR-ESI–MS *m*/*z* 397.2015 [M + H]^+^ (calcd for C_24_H_28_O_5_ + H, 397.2010). IR (KBr) *ν*_max_ 2960, 1758, 1717, 1635, 679 cm^−1^; ^1^H (500 MHz, CDCl_3_) and ^13^C NMR (125 MHz, CDCl_3_) data see Table 1. (+)-(6*R*,7*R*,3′*R*,6′*R*,7′*R*,8′*S*)-**1**: [*α*]_D_^25^ + 17 (*c* 0.08, MeOH); ECD (MeOH) *λ*_max_ (Δ*ε*) 195 (−11.6), 226 (−0.6), 291 (+11.3) nm. (−)-(6*S*,7*S*,3′*S*,6′*S*,7′*S*,8′*R*)-**1**: [*α*]_D_^25^ − 21 (*c* 0.07, MeOH); ECD (MeOH) *λ*_max_ (Δ*ε*) 195 (+15.3), 219 (+2.0), 256 (−8.3) nm.

Compound **2**: colorless gum; UV (MeOH) *λ*_max_ (log *ε*) 274 (4.40) nm; HR-ESI–MS *m*/*z* 414.2271 [M + NH_4_]^+^ (calcd for C_24_H_28_O_5_ + NH_4_, 414.2275). IR (KBr) *ν*_max_ 3437, 2958, 2934, 2872, 1772, 1710, 1674, 1461 cm^−1^; ^1^H (500 MHz, CDCl_3_) and ^13^C NMR (125 MHz, CDCl_3_) data see Table 1. (+)-(6*R*,7*R*,9*R,*3ʹa*R*,6ʹ*R*)-**2**: [*α*]_D_^25^ + 246 (*c* 0.18, MeOH); ECD (MeOH) *λ*_max_ (Δ*ε*) 202 (+24.8), 238 (−4.5), 261 (+2.7), 292 (−10.2) nm. (−)-(6*S*,7*S*,9*S,*3ʹa*S*,6ʹ*S*)-**2**: [*α*]_D_^25^ − 346 (*c* 0.13, MeOH); ECD (MeOH) *λ*_max_ (Δ*ε*) 202 (−27.3), 238 (+6.7), 261 (−1.6), 292 (+12.7) nm.

Compound **3**: yellow oil; HR-ESI–MS *m/z* 205.0861 [M + H]^+^ (calcd for C_24_H_28_O_5_ + H, 205.0859). IR (KBr) *ν*_max_ 3182, 1721, 1670, 1620, 1589 cm^−1^; ^1^H (500 MHz, methanol-*d*_4_) and ^13^C NMR (125 MHz, methanol-*d*_4_) data see Table 1.

### ECD calculation

The theoretical calculations of compounds **1** and **2** were calculated employing the Gaussian program package. Conformational analysis was performed using CONFLEX software. Conformations with a Boltzmann population exceeding 2% were chosen for optimization and spectral calculation. The theoretical calculation of ECD was performed using Time Dependent Density Functional Theory (TDDFT) at B3LYP/6-31G (d,p) (compound **1**) and CAM-B3LYP/DGDZVP (compound **2**) level. The ECD spectra of the compounds **1** and **2** were obtained by considering the Boltzmann distribution of each geometric conformation [21].

### NO inhibitory assay

The NO inhibitory assay was carried out as described previously [20]. For details, see the Supporting Information.

### Statistical analysis

The statistical analysis was carried out as described previously [20].

## Plausible biosynthetic pathway for 1 and 2

**Scheme 1:**
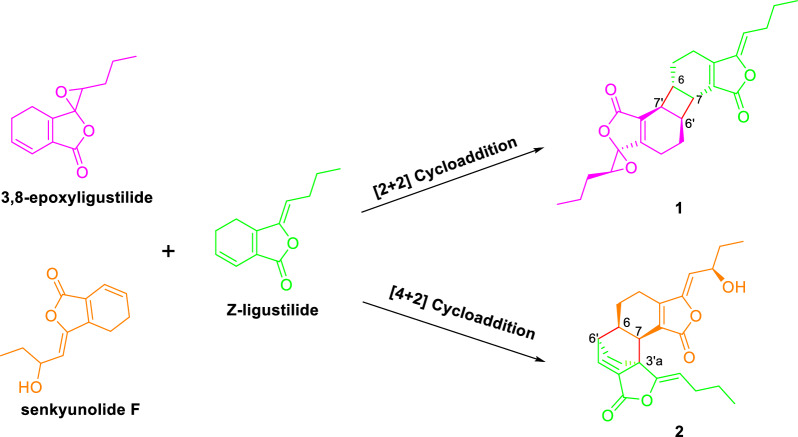
Plausible biosynthetic pathway of compounds **1** and **2**

Phthalide dimers with different linkages are generated by [4 + 2] or [2 + 2] cycloaddition of two monomeric phthalate units. As for **1** and **2**, they might be formed by cycloaddition reaction between *Z*-ligustilide and the molecule of phthalide monomer (3,8-epoxyligustilide [22] and senkyunolide F [23]) (Scheme 1).

## Conclusion

In conclusion, three pairs of enantiomeric phthalides characterized as [2 + 2] and [4 + 2] cycloadducts and a phthalide monomer were obtained from the rhizomes of *A. sinensis*. The racemates were further separated by chiral column and all of them were evaluated for their inhibitory effects on LPS-induced NO production. Interestingly, the current results demonstrated that the phthalide dimers have more potential anti-inflammatory activity than that of monomeric phthalides. The discovery of **1**–**3** are enriching supplement for the diversity of the phthalides category and provides a new insight for their biosynthetic, synthetic, and pharmacological investigation.

## Supplementary Information

**Additional file 1.** 1D and 2D NMR, HR-ESI-MS, and UV data of compounds **1**–**3**, the chiral separation of **1** and **2**.

## Data Availability

All data generated or analyzed during this study are included in this published article and its Additional file 1.
