# Supplementary material for: Phthalide mono- and dimers from the rhizomes of Angelica sinensis and their anti-inflammatory activities
Source: Nat Prod Bioprospect. 2025 Apr 24;15(1):26. doi: 10.1007/s13659-025-00512-z (PMC12021743; doi:10.1007/s13659-025-00512-z)
Supplement: Supplementary file 1 — Additional file 1. 1D and 2D NMR, HR-ESI-MS, and UV data of compounds 1–3, the chiral separation of 1 and 2. [file 13659_2025_512_MOESM1_ESM.pdf]

Supporting Information

Phthalide mono- and dimers from the rhizomes of *Angelica sinensis* and their anti-inflammatory activities

Hongyan Wen[a, b], Sheng Li[b], Yu Zhang [b]*

[a] *College of Ethnic Medicine, Yunnan University of Chinese Medicine, Kunming 650500, China*

[b] *State Key Laboratory of Phytochemistry and Natural Medicines, Kunming Institute of Botany, Chinese Academy of Sciences, Kunming 650201, China*

Table of Contents

Experimental section

1. Extraction and isolation.
2. Chiral separation.
3. NO inhibitory assay.
4. Statistical analysis.

Figure S1 The effect of compounds **1-4** on macrophage viability in RAW 264.7.

NMR, HR-ESI-MS, IR, UV, ECD Spectra

For compound 1:

Figure S1.1 ^{1}H NMR spectrum of compound **1** in $CDCl_3$ (500 MHz).

Figure S1.2 ^{13}C NMR spectrum of compound **1** in $CDCl_3$ (125 MHz).

Figure S1.3 DEPT spectrum of compound **1** in $CDCl_3$ (125 MHz).

Figure S1.4 ^{1}H- ^{1}H COSY spectrum of compound **1** in $CDCl_3$.

Figure S1.5 HSQC spectrum of compound **1** in $CDCl_3$.

Figure S1.6 HMBC spectrum of compound **1** in $CDCl_3$.

Figure S1.7 ROESY spectrum of compound **1** in $CDCl_3$.

Figure S1.8 HR-ESI-MS spectrum of compound **1.**

Figure S1.9 IR spectrum of compound **1.**

FigureS1.10 ECD spectrum of compound (+)-**1** in MeOH.

Figure S1.11 UV spectrum of compound (+)-**1**.

Figure S1.12 ECD spectrum of compound (−)-**1** in MeOH.

Figure S1.13 UV spectrum of compound (−)-**1**.

For compound 2:

Figure S2.1 ^{1}H NMR spectrum of compound **2** in $CDCl_3$ (500 MHz).

Figure S2.2 ^{13}C NMR spectrum of compound **2** in $CDCl_3$ (125 MHz).

Figure S2.3 DEPT spectrum of compound **2** in $CDCl_3$ (125 MHz).

Figure S2.4 ^{1}H- ^{1}H COSY spectrum of compound **2** in $CDCl_3$.

Figure S2.5 HSQC spectrum of compound **2** in $CDCl_3$.

Figure S2.6 HMBC spectrum of compound **2** in $CDCl_3$.

Figure S2.7 ROESY spectrum of compound **2** in $CDCl_3$.

Figure S2.8 HR-ESI-MS spectrum of compound **2**.

Figure S2.9 IR spectrum of compound **2.**

Figure S2.10 ECD spectrum of compound **(+)-2** in MeOH.

Figure S2.11 UV spectrum of compound **(+)-2**.

Figure S2.12 ECD spectrum of compound **(−)-2** in MeOH.

Figure S2.13 UV spectrum of compound **(−)-2**.

For compound 3:

Figure S3.1 ^{1}H NMR spectrum of compound **3** in methanol-d_4 (500 MHz)

Figure S3.2 ^{13}C NMR spectrum of compound **3** in methanol-d_4 (125 MHz).

Figure S3.3 ^{1}H- ^{1}H COSY spectrum of compound **3** in methanol-d_4.

Figure S3.4 HSQC spectrum of compound **3** in methanol-d_4.

Figure S3.5 HMBC spectrum of compound **3** in methanol-d_4.

Figure S3.6 ROESY spectrum of compound **3** in methanol-d_4.

Figure S3.7 HR-ESI-MS spectrum of compound **3**.

Figure S3.8 IR spectrum of compound **3**.

1. Extraction and isolation

The dried roots of *A. sinensis* (15.0 kg) were extracted with 80% EtOH (3 × 7 d) under reflux. The extract (8.5 kg) was evaporated under reduced pressure to yield a residue that was later suspended in H_2O, and then partitioned using Amberlite D101 with MeOH-H_2O (0:100, 90:10, 100:0) to obtain three fractions (Fr. A-C). Fr. C (575.0 g) was subjected to gradient elution on a silica gel chromatography column with petroleum ether-acetone (40:1, 20:1, 10:1, 5:1, 1:1) to give three subfractions (Fr. C-I-III). The Fr. C-III (20 g) was divided into three fractions (Fr. C-IIIa-c) by MCI gel column eluting with a gradient from MeOH–H_2O (10:90 → 100:0), Fr. C-IIIa was purified using Sephadex LH-20 (CH_2Cl_2/MeOH: 1:1) and separated by semipreparative HPLC using a Waters X-Bridge C18 (10 × 150 mm, 5 μm) column with MeCN/H_2O (55:45, 3 ml/min) to afford **1** (4.5 mg, t_R=10.0 min) and **4** (9.5 mg, t_R=26.0 min). Compounds **2** (45.4 mg, t_R=38.0 min) and **3** (3.4 mg, t_R=33.0 min) were obtained from Fr. C-Ib by Sephadex LH-20 (CH_2Cl_2/MeOH: 1:1) and semi-preparative high performance liquid chromatography (HPLC) using a Waters X-Bridge C18 (10 × 150 mm, 5 μm) column with MeCN/H_2O (40:60, 3 ml/min).

2. Chiral separation

2.1 The chiral HPLC separation of 1 (4.5 mg).

Chromatographic conditions:

(1) Column: ChiralPak AD-H (10 × 250 mm, i.d. 5 μm)

(2) Mobile phase: n-hexane/isopropanol (94:6)

(3) UV detection: 210 and 254 nm

(4) Flow rate: 1 mL/min

(5) Retention time: (+)-**1** (0.8 mg, 36.5 min), (−)-**1** (0.7 mg, 40.0 min)

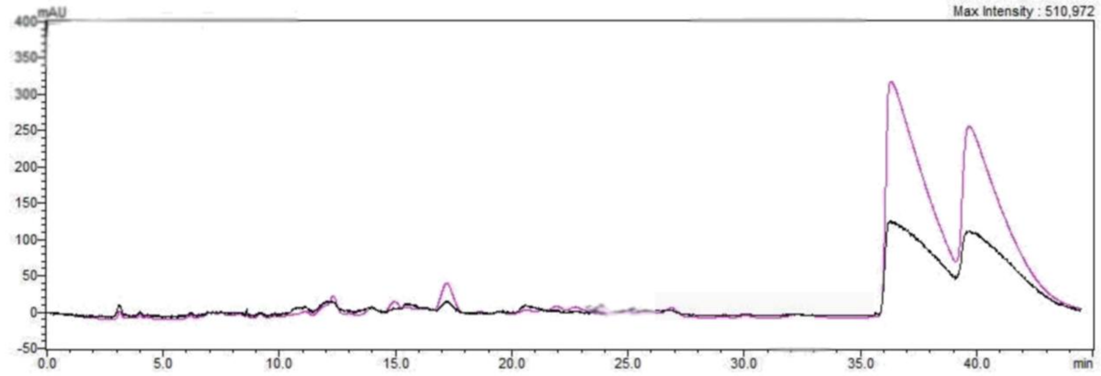

2.2 The chiral HPLC separation of 2 (13.0 mg).

Chromatographic conditions:

(1) Column: ChiralPak AD-H (10 × 250 mm, i.d. 5 μm)

(2) Mobile phase: n-hexane/2-isopropanol (90:10)

(3) UV detection: 210 and 254 nm

(4) Flow rate: 1 mL/min

(5) Retention time: (+)-**2** (1.3 mg, 29.0 min), (−)-**2** (1.8 mg, 34.0 min)

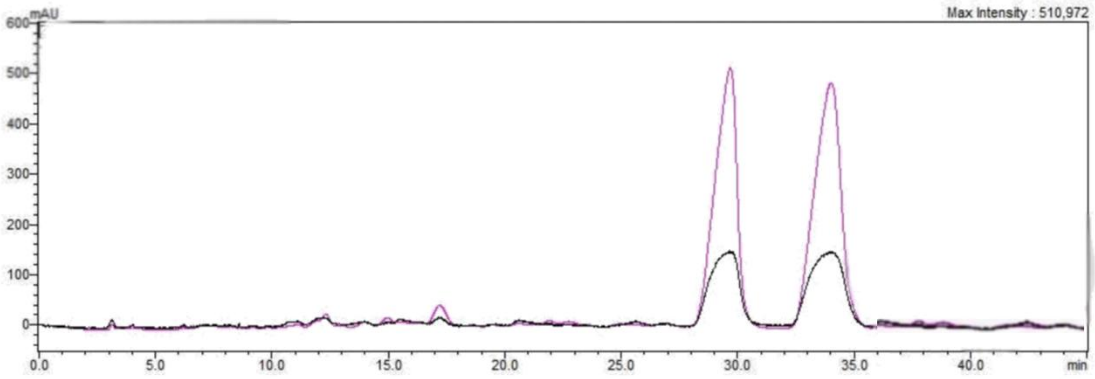

2.3 The chiral HPLC separation of 4 (9.5 mg).

Chromatographic conditions:

(1) Column: ChiralPak AD-H (10 × 250 mm, i.d. 5 μm)

(2) Mobile phase: n-hexane/2-isopropanol (92:8)

(3) UV detection: 210 and 254 nm

(4) Flow rate: 1 mL/min

(5) Retention time: (+)-**4** (2.3 mg, 47.0 min), (−)-**4** (1.5 mg, 50.0 min)

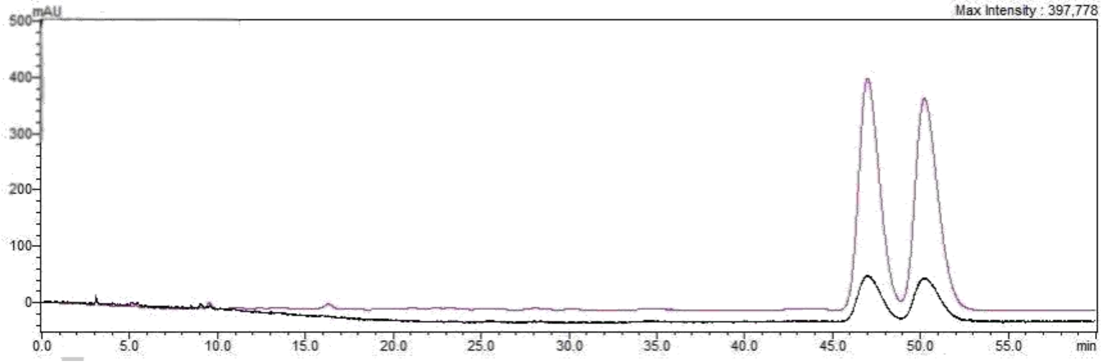

3. NO inhibitory assay

The inhibitory activities against NO production of all the isolates were elucidated by preventing the expression of NO production in LPS-induced RAW 246.7 mouse macrophages. The RAW264.7 cells were cultivated in DMEM medium containing with 10% FBS, 100 U/ mL penicillin, and 100 μg/mL streptomycin at 37 °C with 5% CO_2. Firstly, the cells were cultured in 96-well plates (80000 cells/well) and incubated for 4 h. Then, LPS at a concentration of 1 μg/mL was added together with the different concentrations of tested compounds (dissolved in Dimethyl Sulphoxide). After 24 h, the concentration of nitrite in the culture supernatant was measured using Griess reagent to determine NO production. N-Monomethyl-*L*-Arginine (*L*-NMMA, Sigma-Aldrich, USA) was used as the positive control. The cell viability of compounds **1**-**4** on RAW264.7 cells were elucidated by the standard MTS assay. 20 μL MTS reagent (Promega Biotech Co., Ltd, Beijing, China) was added into each well after NO assays, which was further incubated for 1.5 h. Absorbance was measured by

a microplate reader at 490 nm.

4. Statistical analysis

All the data and results presented underwent validation through at least three independent experiments. Statistical analysis was performed using GraphPad Prism 8 software. The one-way ANOVA (analysis of variance) was performed using the Dunnett's post hoc test for comparison between the treated group and control group, and the values were expressed as mean ± standard deviation (SD). A P-value of < 0.05 was considered statistically significant throughout the study.

Figure S1 The effect of compounds **1-4** on macrophage viability in RAW 264.7.

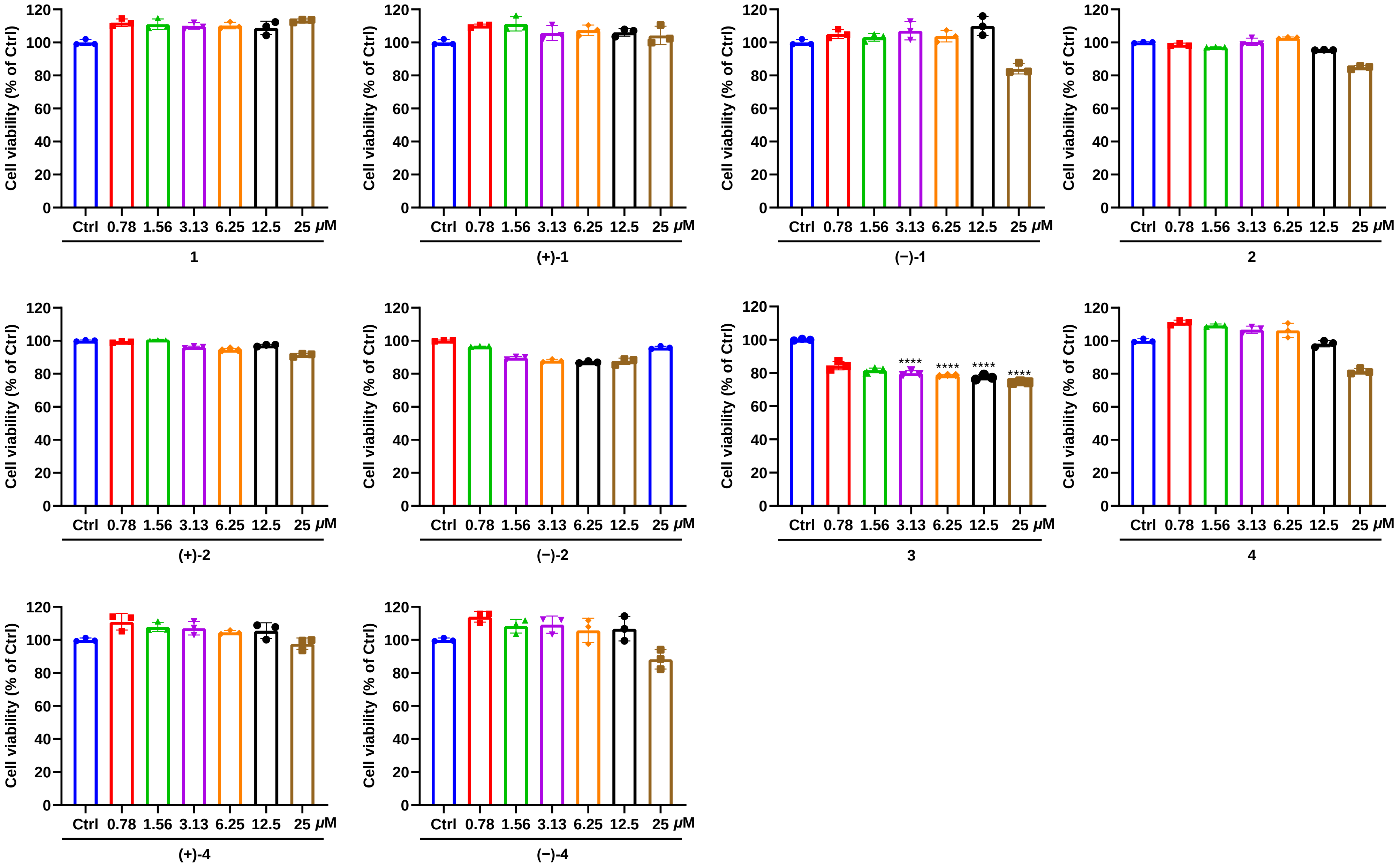

Figure S1.1 ¹H NMR spectrum of compound 1 in $CDCl_3$ (500 MHz).

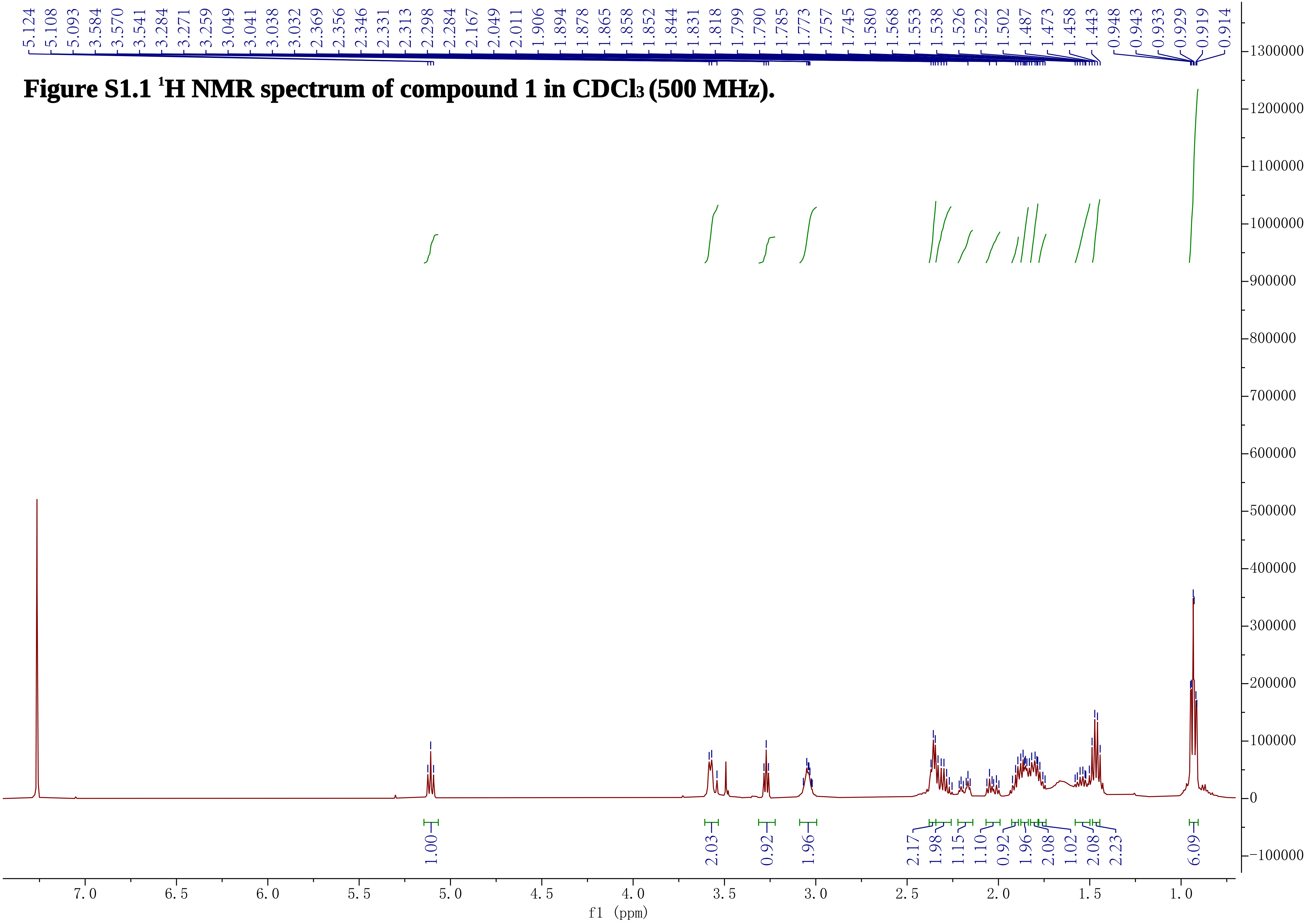

Figure S1.2 ^{13}C NMR spectrum of compound 1 in $CDCl_3$ (125 MHz).

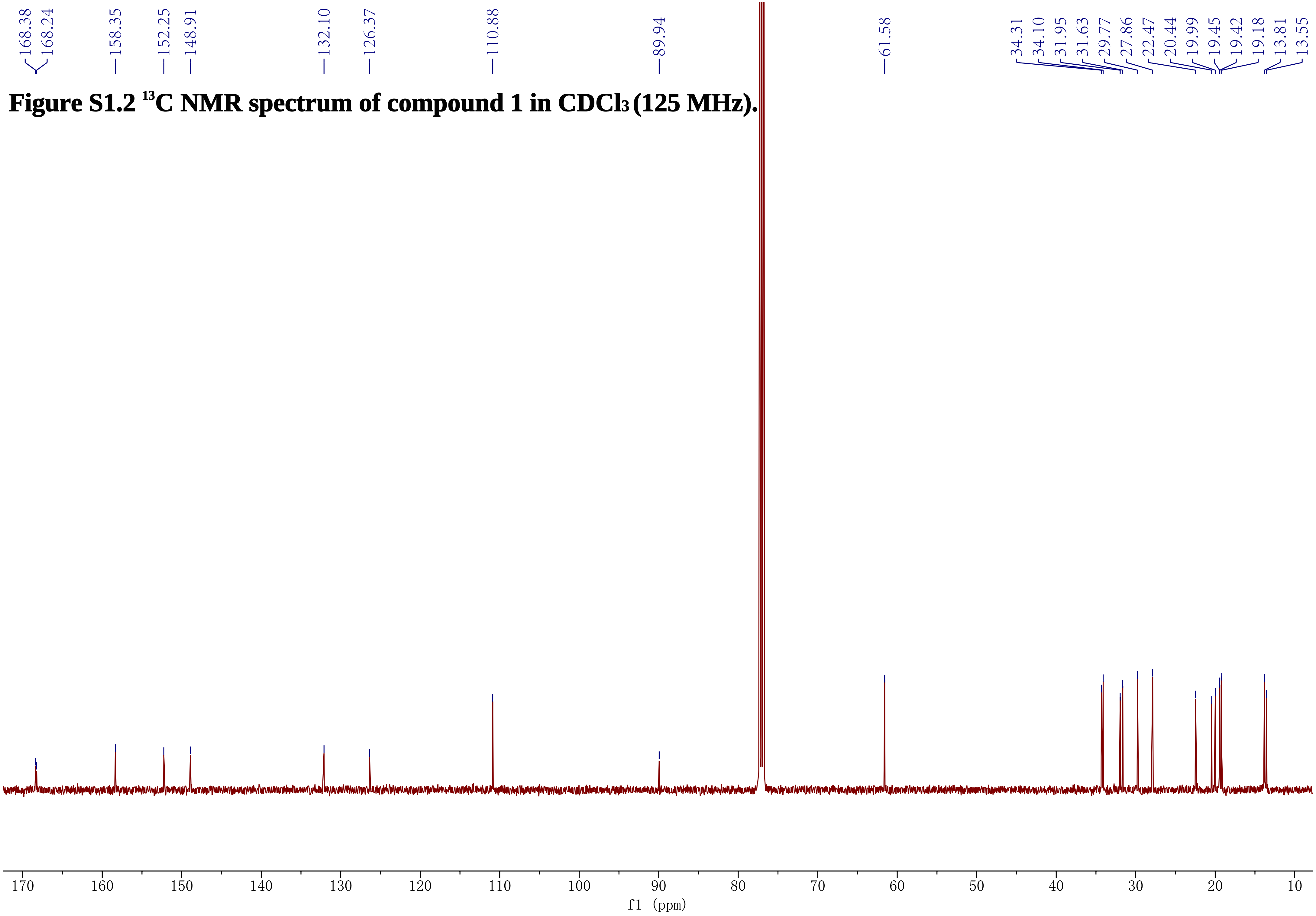

Figure S1.3 DEPT spectrum of compound 1 in $CDCl_3$ (125 MHz).

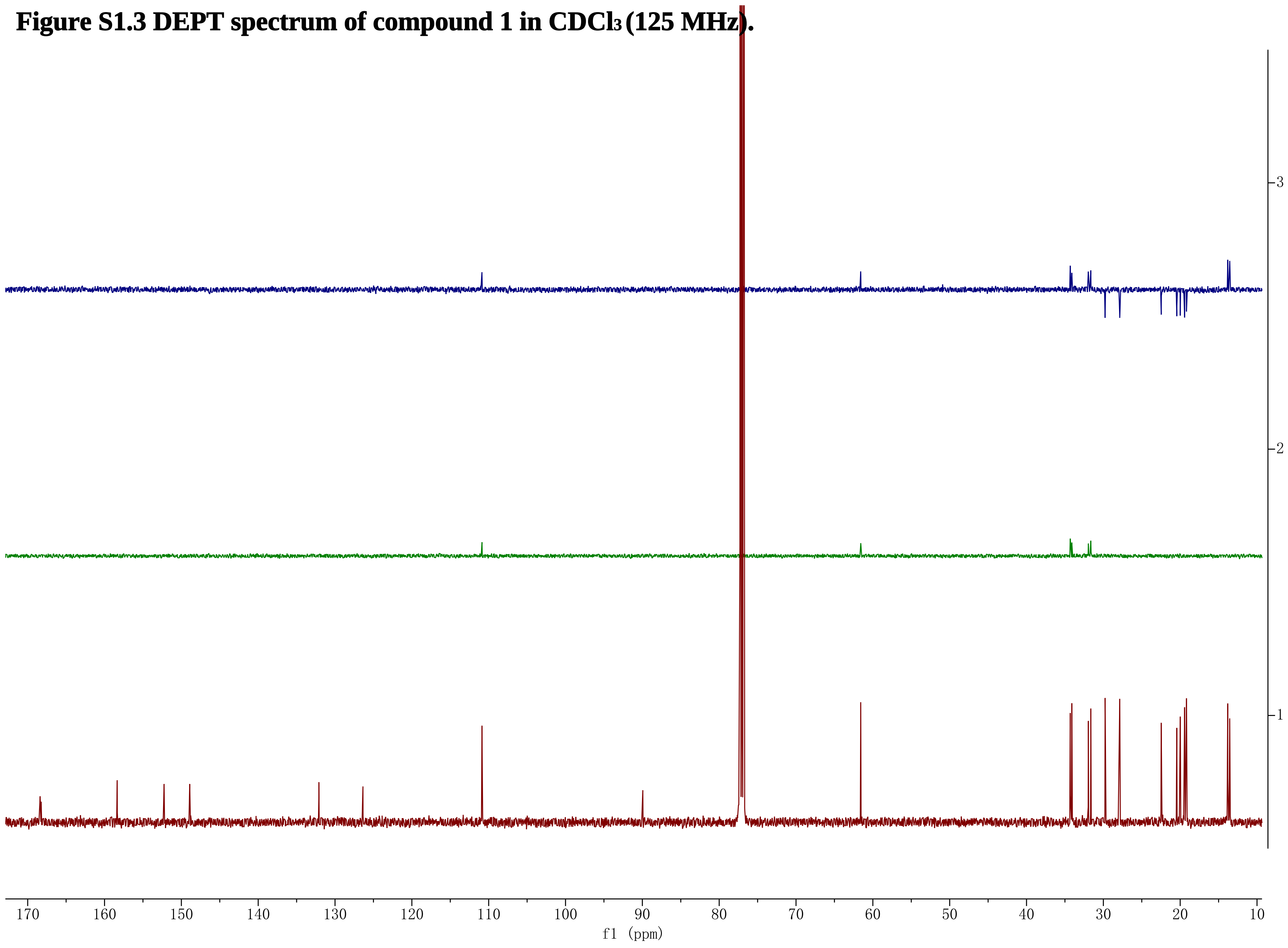

Figure S1.4 ¹H- ¹H COSY spectrum of compound 1 in $CDCl_3$.

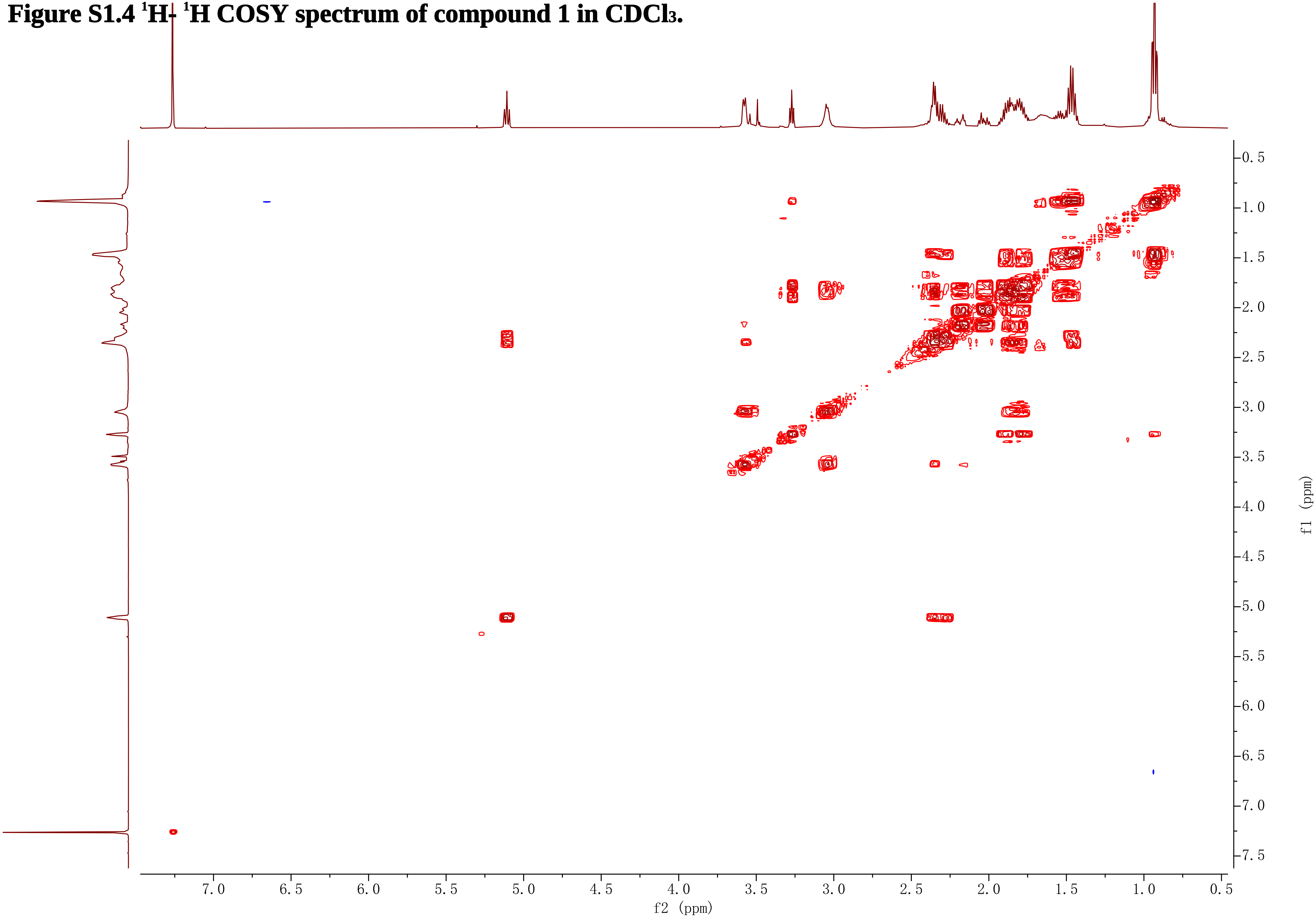

Figure S1.5 HSQC spectrum of compound 1 in CDCl₃.

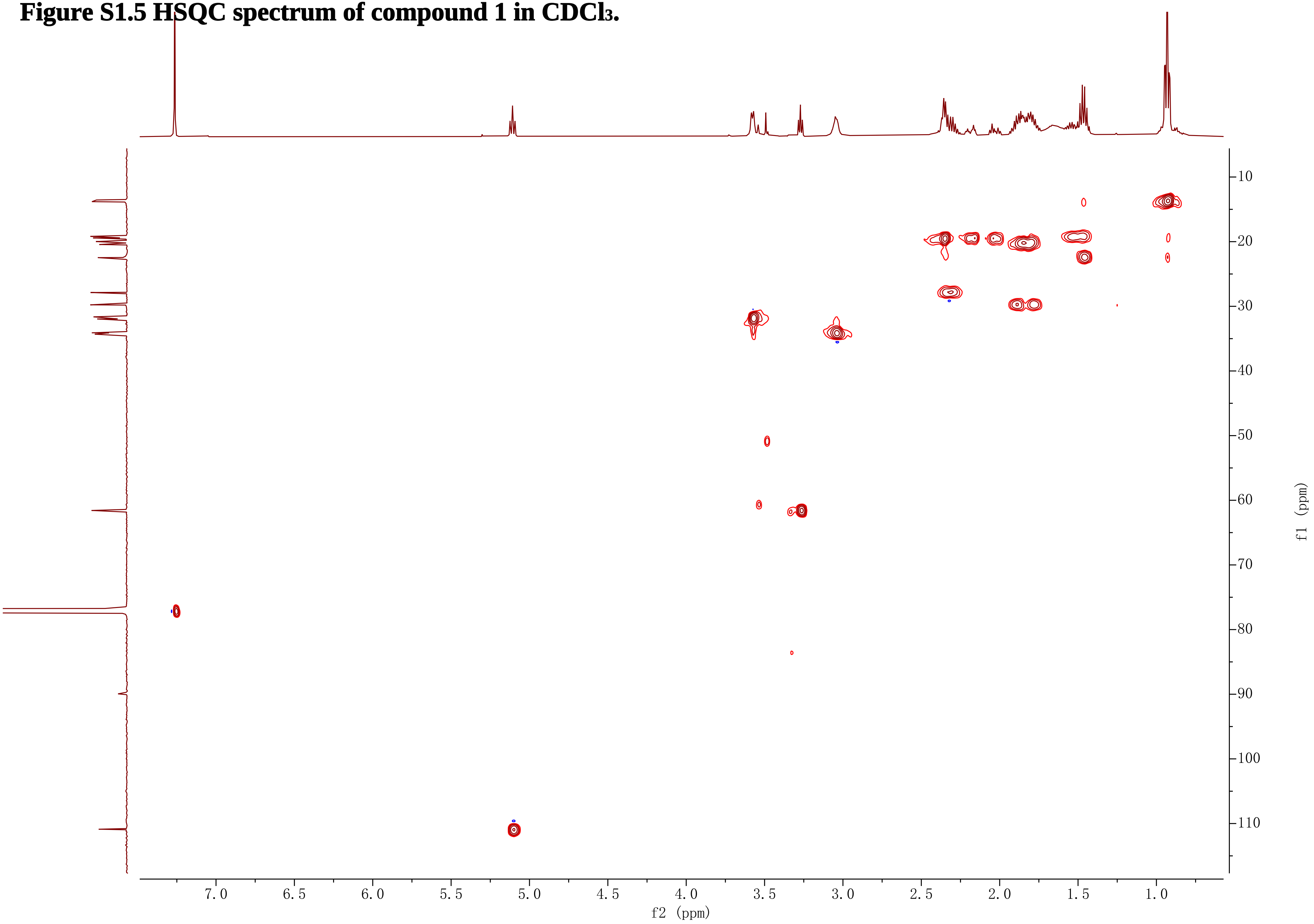

Figure S1.6 HMBC spectrum of compound 1 in $CDCl_3$.

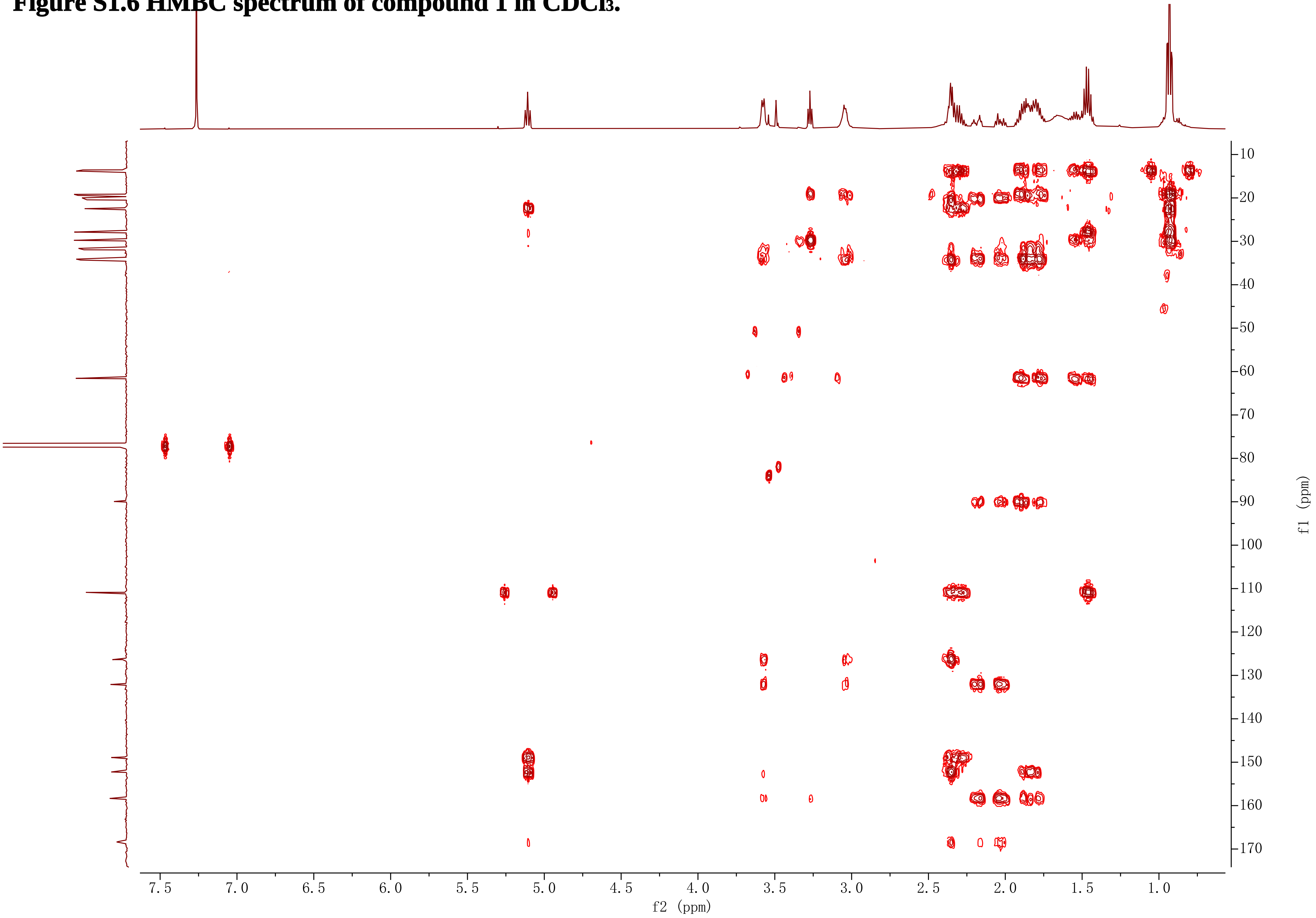

Figure S1.7 ROESY spectrum of compound 1 in $CDCl_3$.

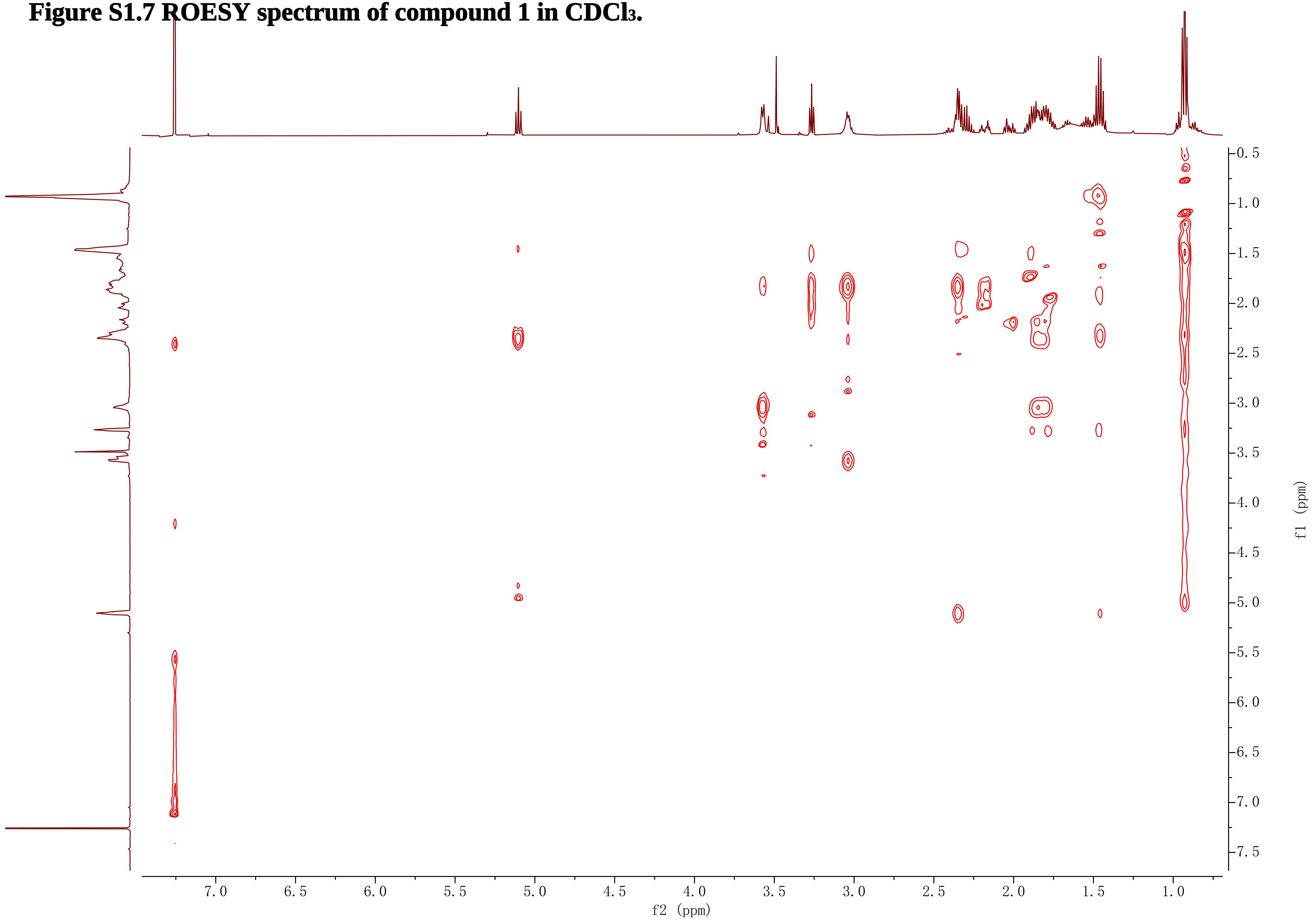

Figure S1.8 HR-ESI-MS spectrum of compound 1.

Qualitative Analysis Report

**Data Filename**	1.d	**Sample Name**	1
**Sample Type**	Sample	**Position**	P1-F1
**Instrument Name**	Instrument 1	**User Name**	
**Acq Method**	s.m	**Acquired Time**	3/5/2024 11:00:27 AM
**IRM Calibration Status**	Success	**DA Method**	PCDL.m
**Comment**			

**Sample Group**		**Info.**
**Acquisition SW Version**	6200 series TOF/6500 series Q-TOF B.05.01 (B5125.2)	

User Spectra

Fragmentor Voltage	Collision Energy	Ionization Mode
135	0	ESI

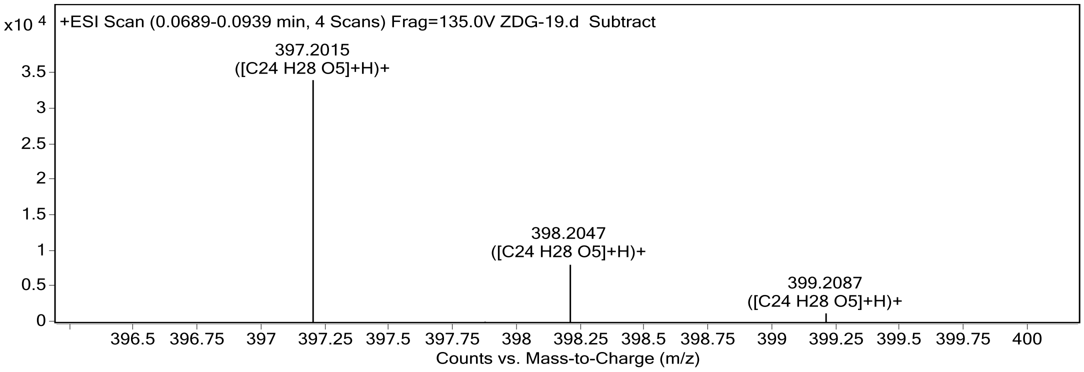

Peak List

m/z	z	Abund	Formula	Ion
103.9558	1	6992.35		
144.9819	1	20218.35		
146.9801	1	10668.23		
397.2015	1	34061	C24 H28 O5	(M+H)+
398.2047	1	8254.98	C24 H28 O5	(M+H)+
414.2274	1	12952.79		
419.1832	1	22978.31		
420.1863	1	5710.74		
815.3778	1	19938.71		
816.3812	1	10057.31		

Formula Calculator Element Limits

Element	Min	Max
C	3	60
H	0	150
O	0	30

Formula Calculator Results

Formula	CalculatedMass	CalculatedMz	Mz	Diff. (mDa)	Diff. (ppm)	DBE
C24 H28 O5	396.1937	397.2010	397.2015	-0.50	-1.26	11.0000

--- End Of Report ---

Figure S1.9 IR spectrum of compound 1

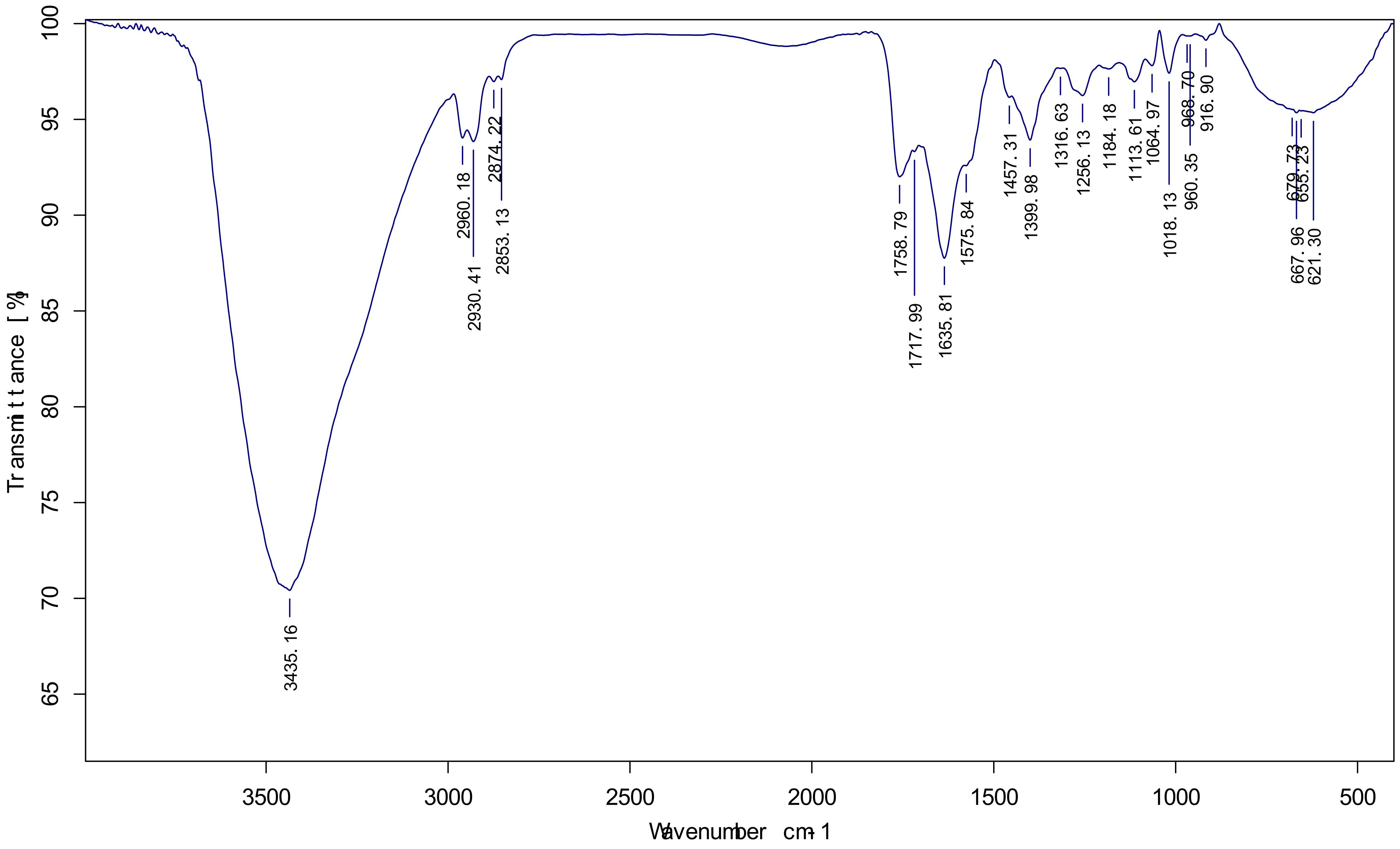

Sample Name: 1
Sample Form: KBr
Path of File: E:\data
Date of Measurement: 2024/10/8

Resolution: 4
Aperture Setting: 6 mm
Number of Background Scans: 16
Number of Sample Scans: 16

Beamsplitter Setting: KBr
Source Setting: MIR
Instrument Type: BRUKER VERTEX 70
Soft Version: OPUS8.1

Figure S1.10 ECD spectrum of compound (+)-1 in MeOH.
（Concentration: 0.6400 mg/mL MeOH）

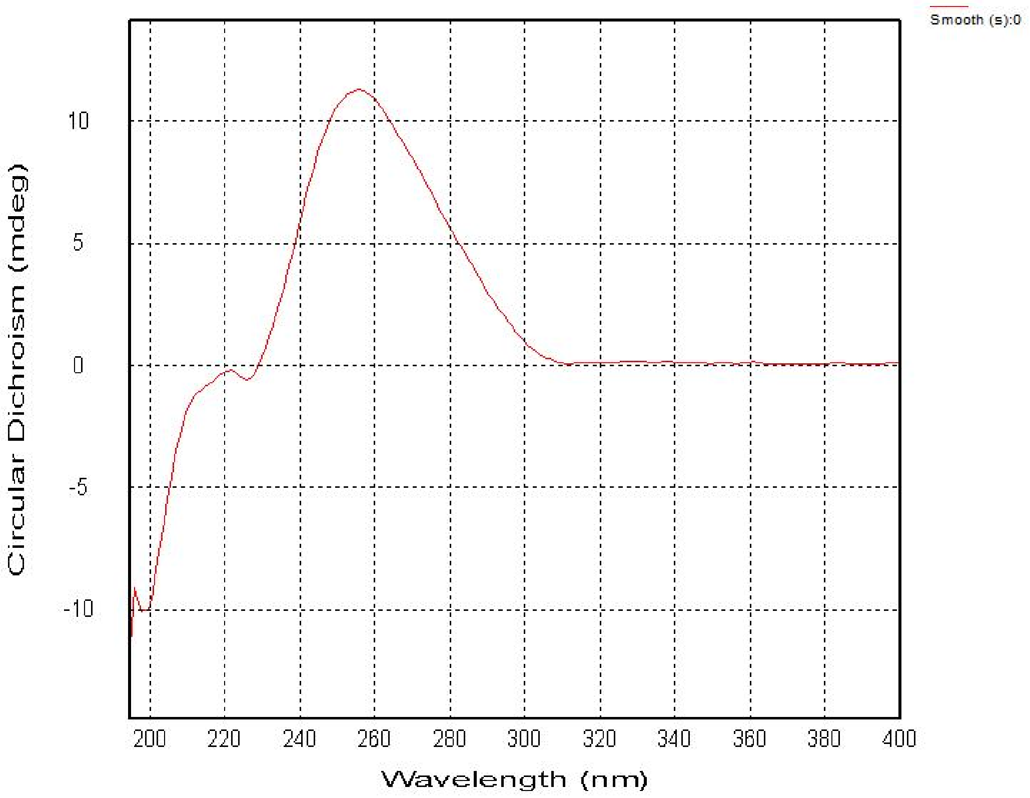

Figure S1.11 UV spectrum of compound (+) -1.

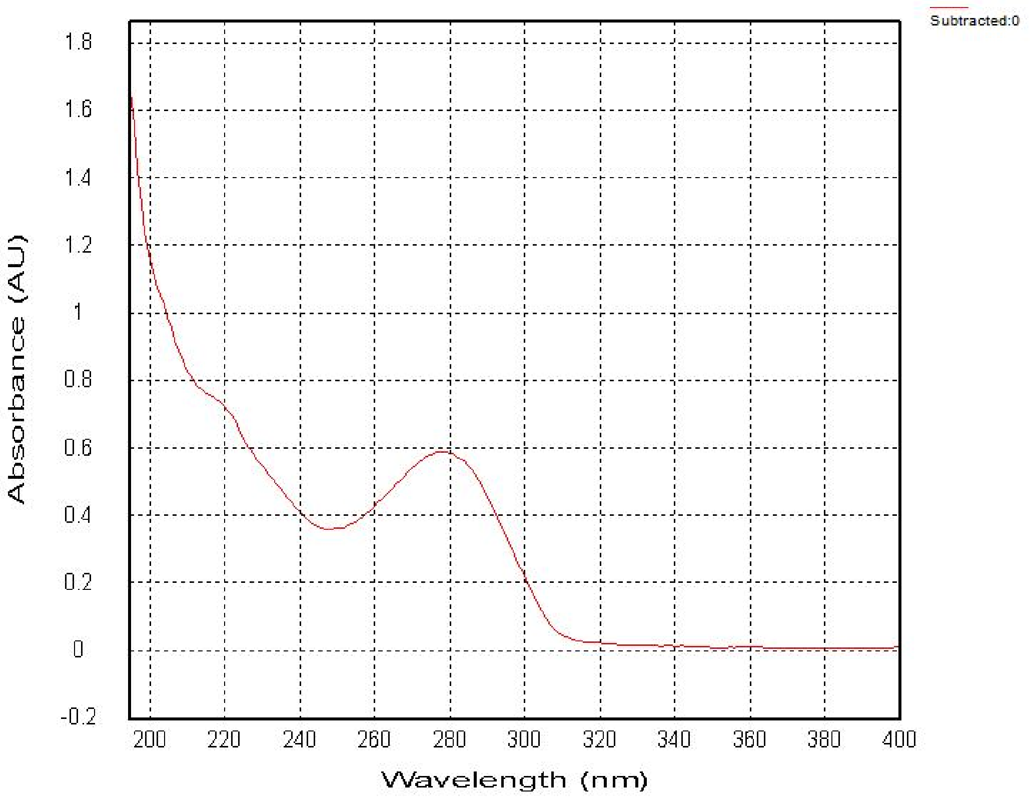

Figure S1.12 ECD spectrum of compound (-)-1 in MeOH.
(Concentration: 0.5040 mg/mL MeOH)

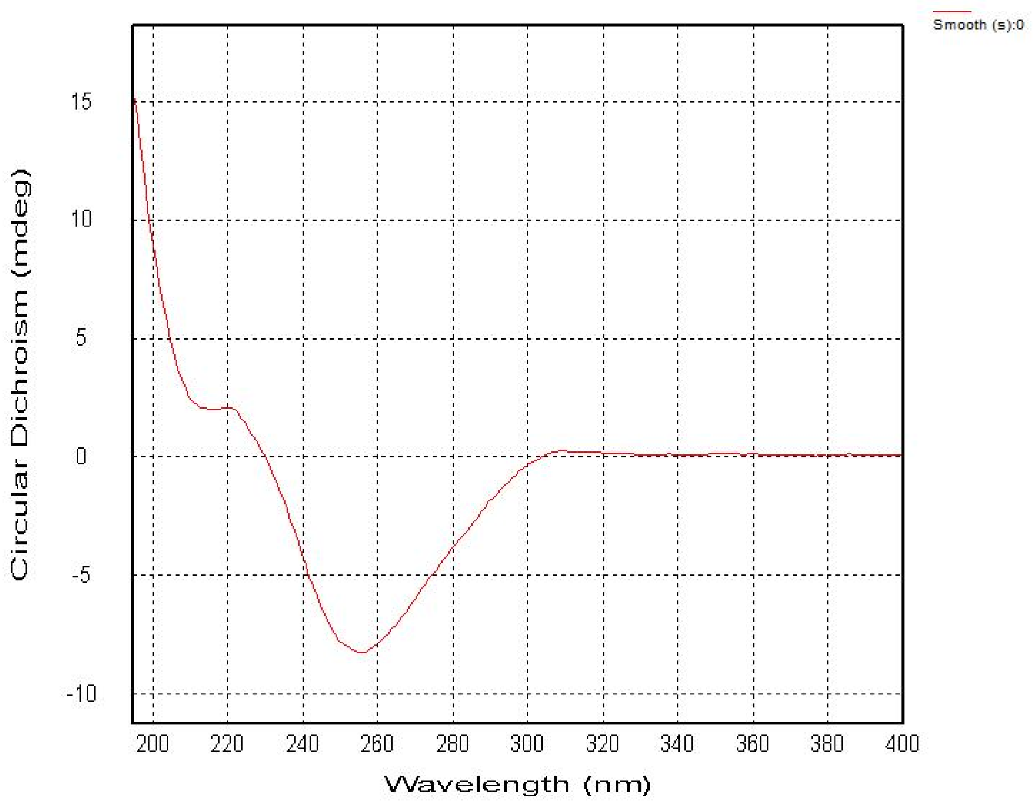

Figure S1.13 UV spectrum of compound (-) -1.

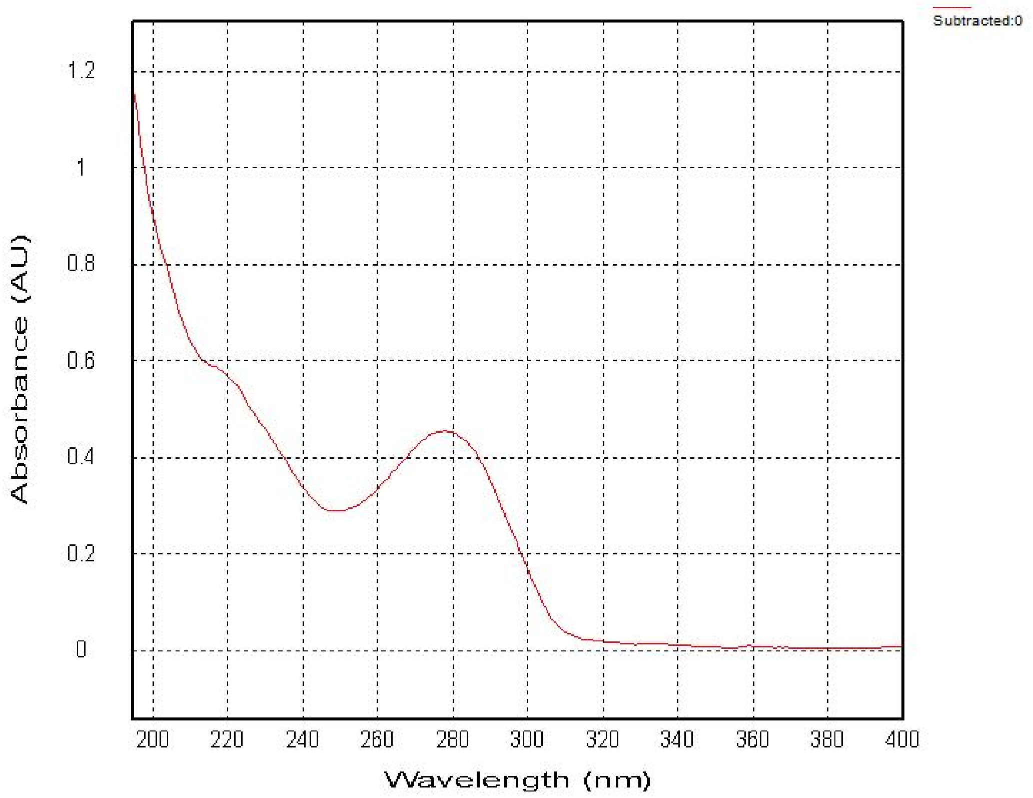

Figure S2.1 ^{1}H NMR spectrum of compound 2 in $CDCl_3$ (500 MHz).

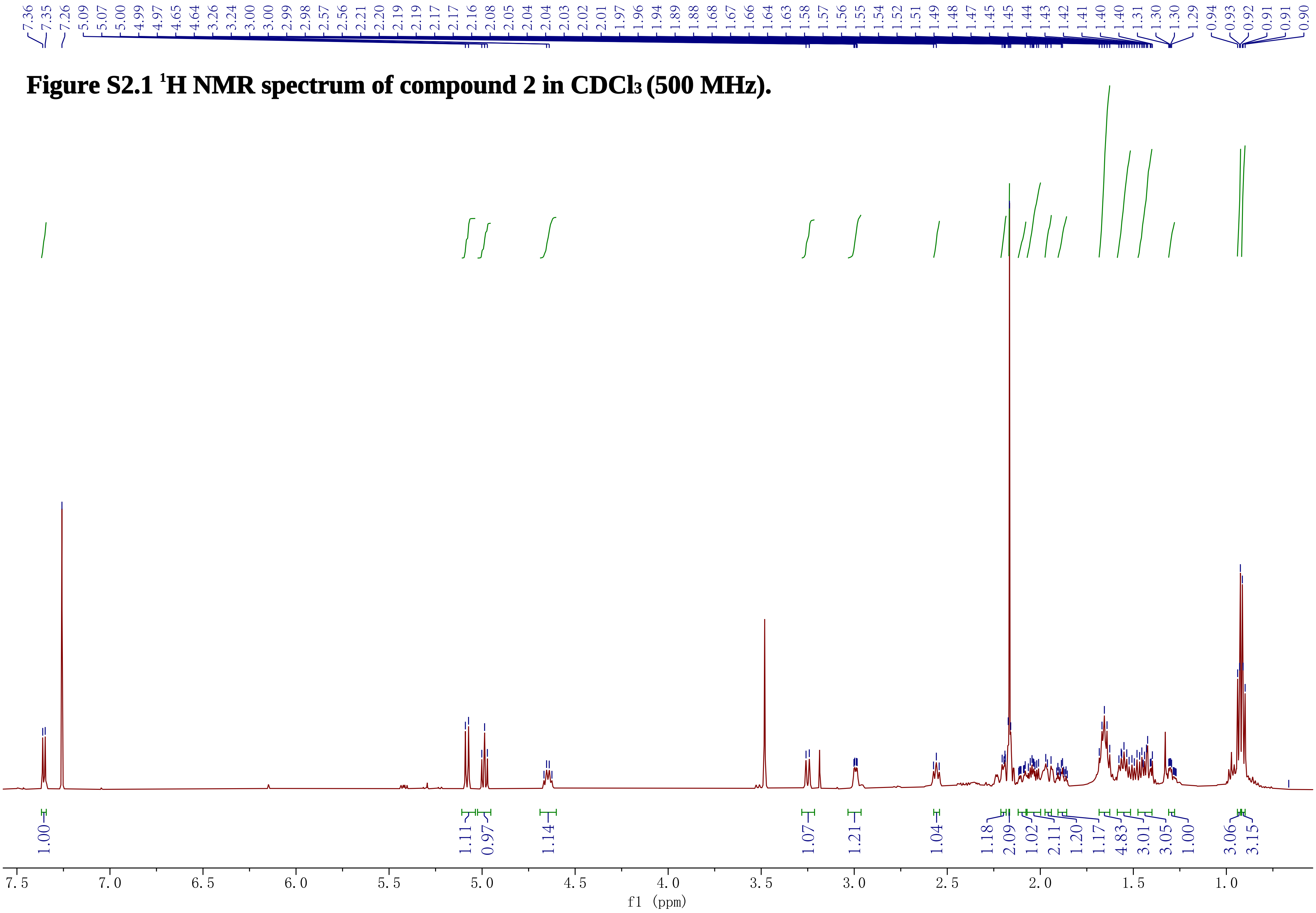

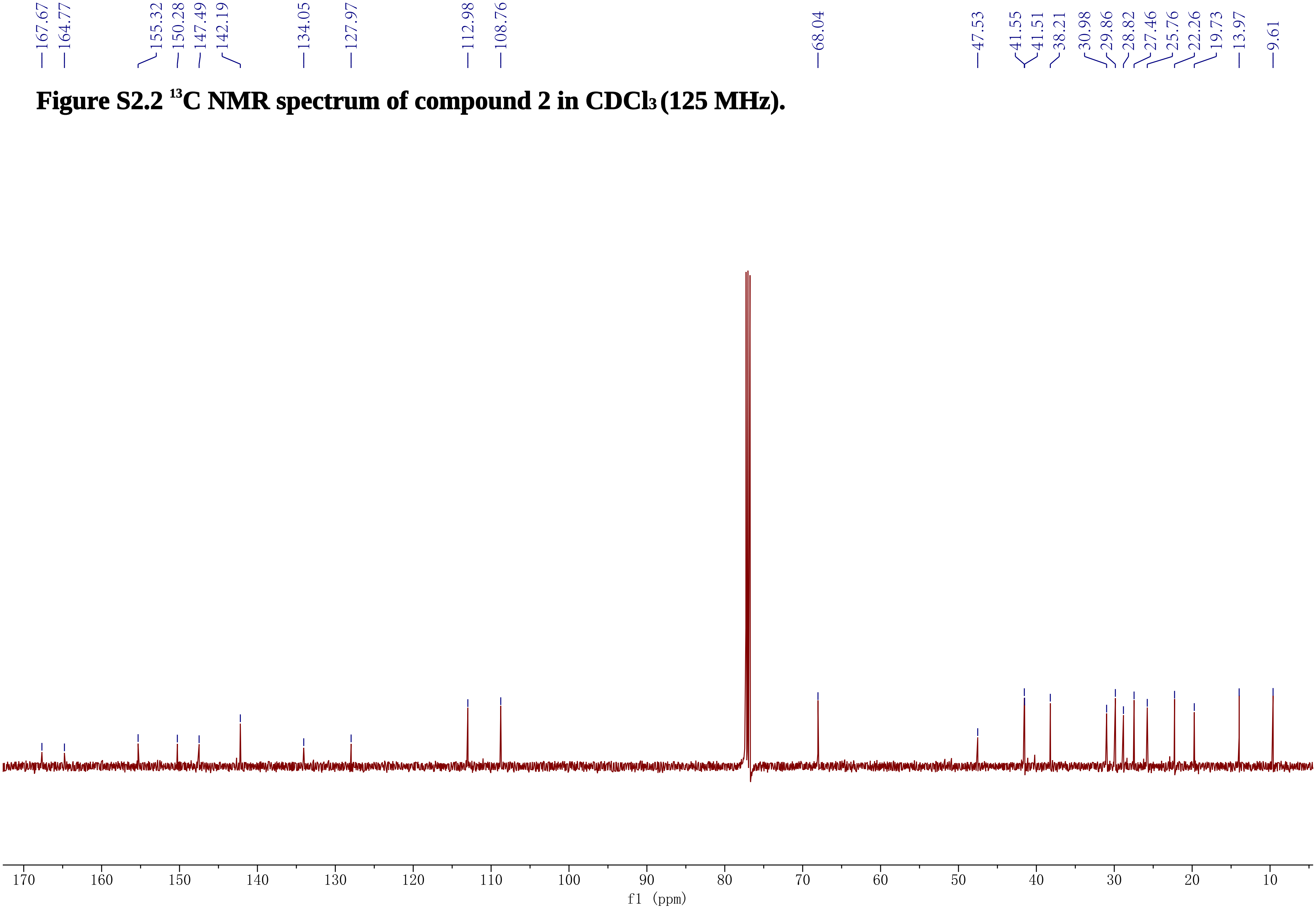

Figure S2.2 ^{13}C NMR spectrum of compound 2 in $CDCl_3$ (125 MHz).

Figure S2.3 DEPT spectrum of compound 2 in $CDCl_3$ (125 MHz).

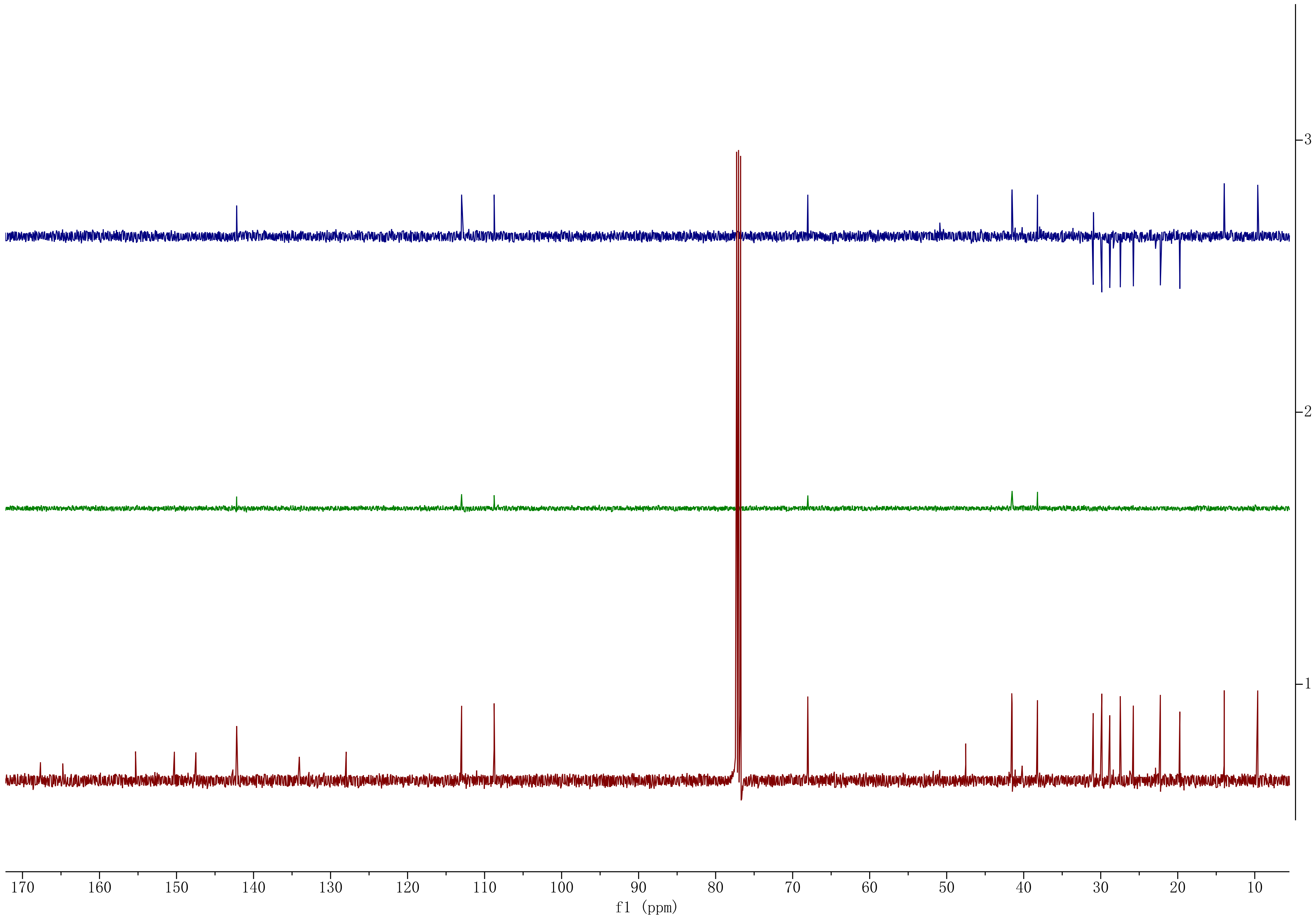

Figure S2.4 ^{1}H- ^{1}H COSY spectrum of compound 2 in CDCl₃.

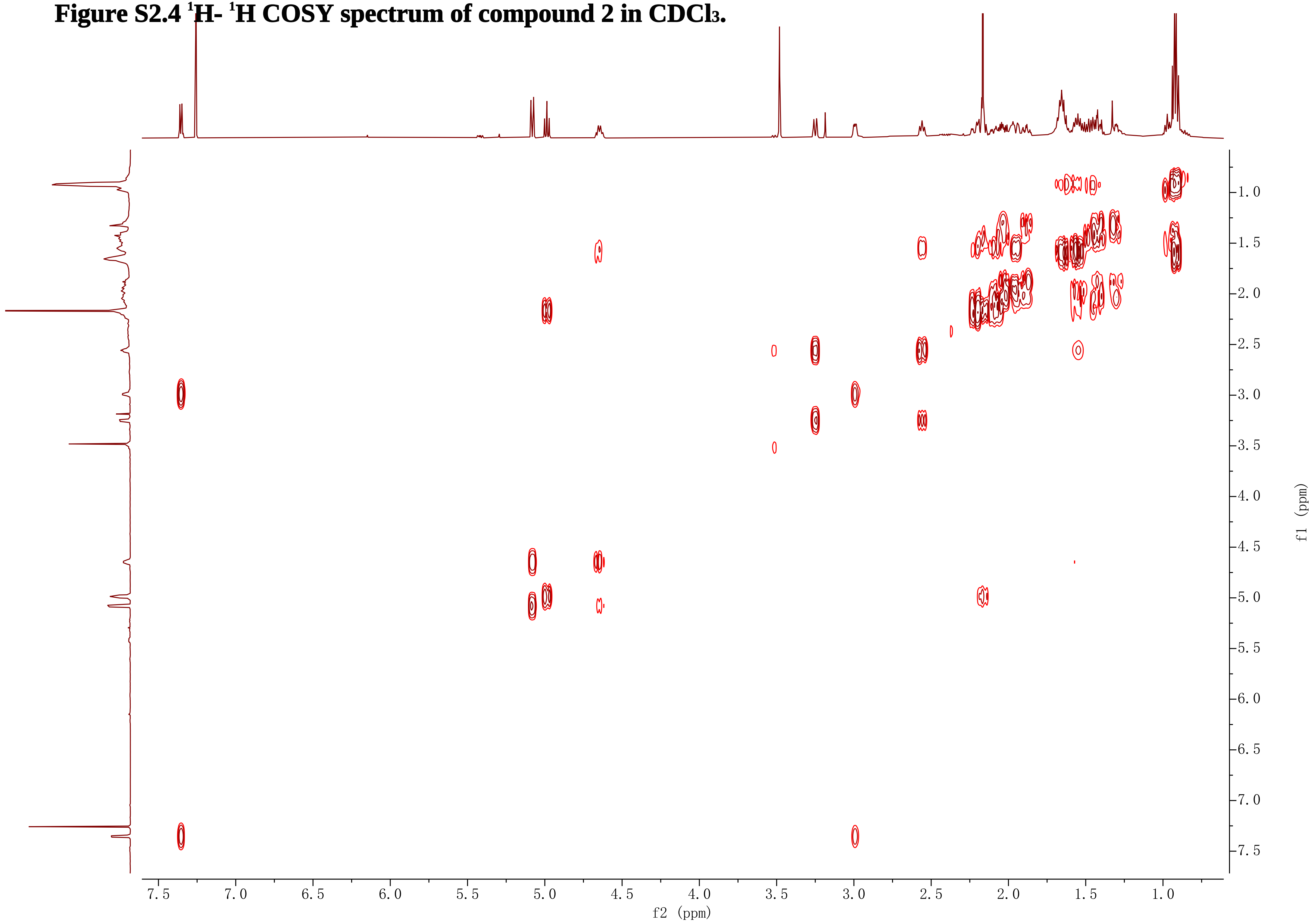

Figure S2.5 HSQC spectrum of compound 2 in $CDCl_3$.

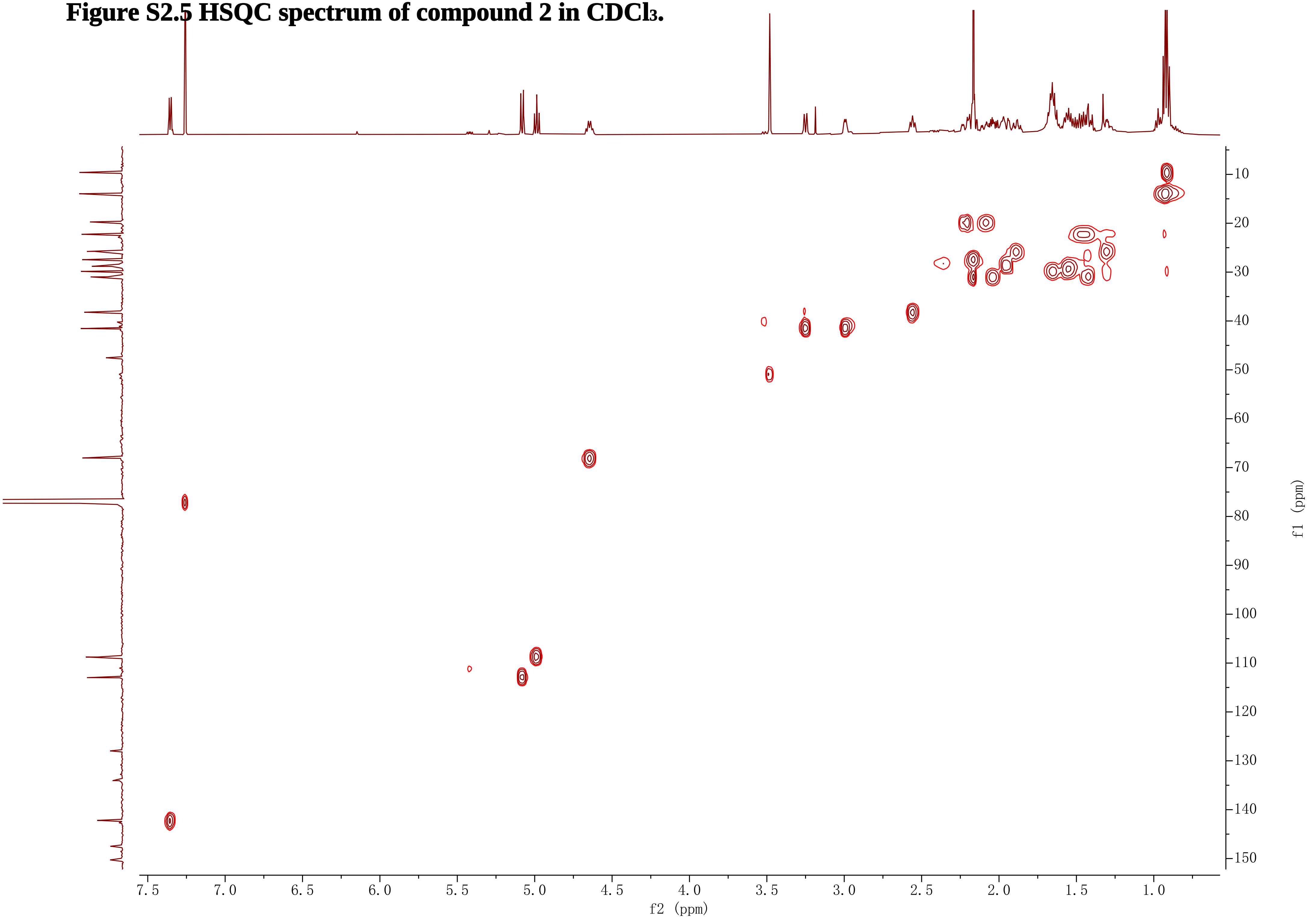

Figure S2.6 HMBC spectrum of compound 2 in $CDCl_3$.

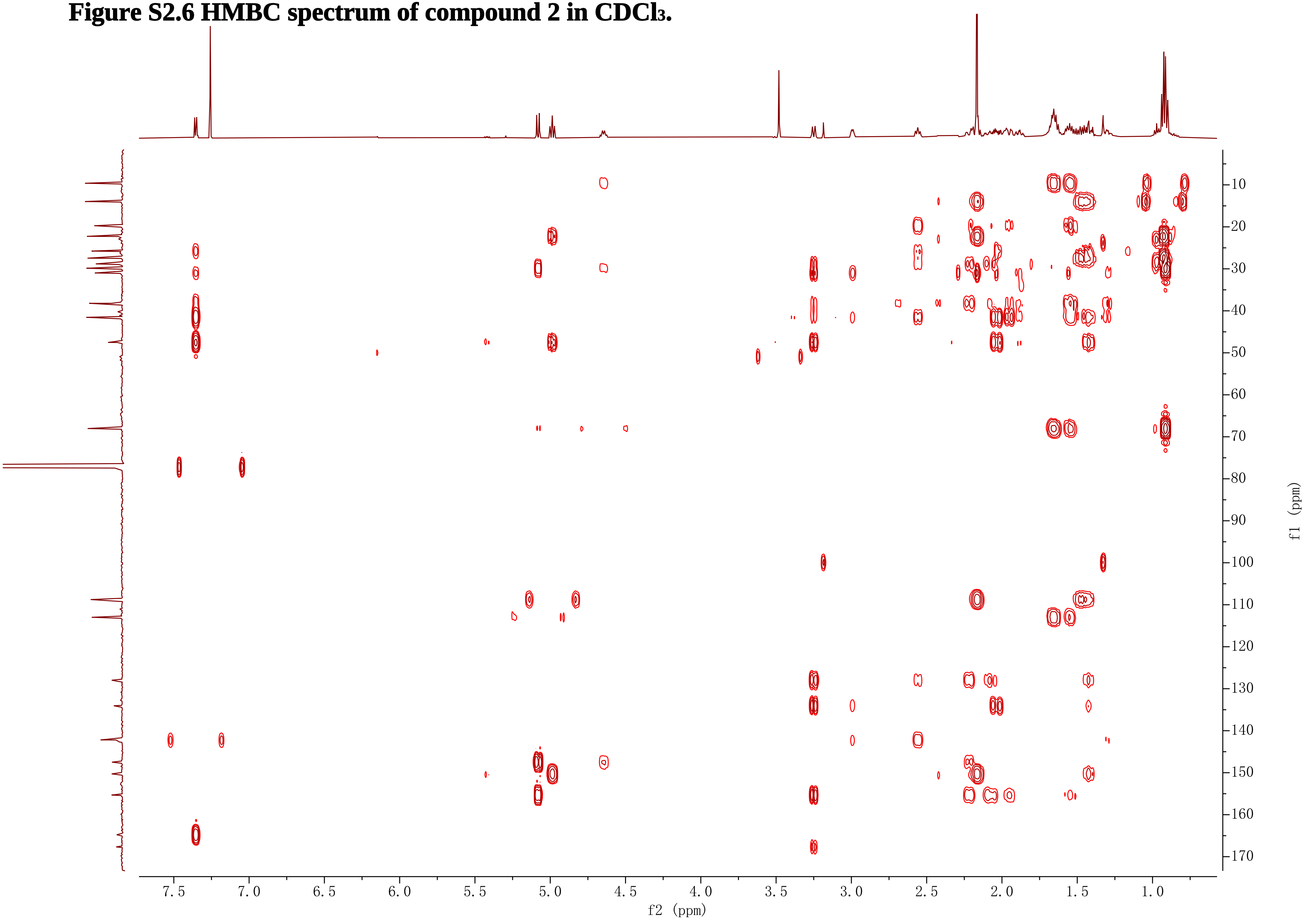

Figure S2.7 ROESY spectrum of compound 1 in $CDCl_3$.

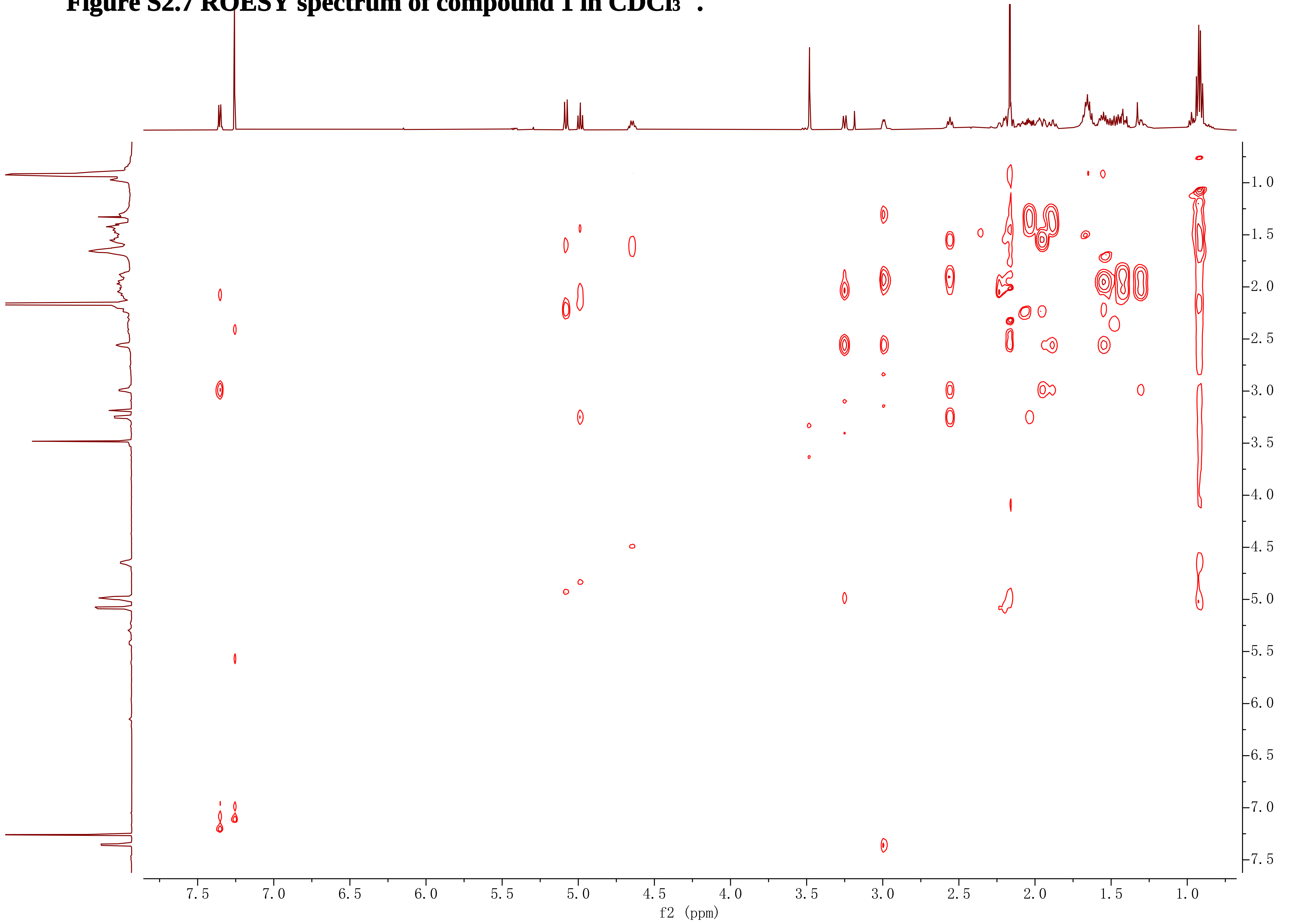

Figure S2.8 HR-ESI-MS spectrum of compound 2.

Qualitative Analysis Report

**Data Filename**	2.d	**Sample Name**	2
**Sample Type**	Sample	**Position**	P1-A4
**Instrument Name**	Instrument 1	**User Name**	
**Acq Method**	s.m	**Acquired Time**	5/27/2024 1:55:51 PM
**IRM Calibration Status**	Success	**DA Method**	PCDL.m
**Comment**			

Sample Group **Info.**
Acquisition SW 6200 series TOF/6500 series
Version Q-TOF B.05.01 (B5125.2)

User Spectra

Fragmentor Voltage	Collision Energy	Ionization Mode
135	0	ESI

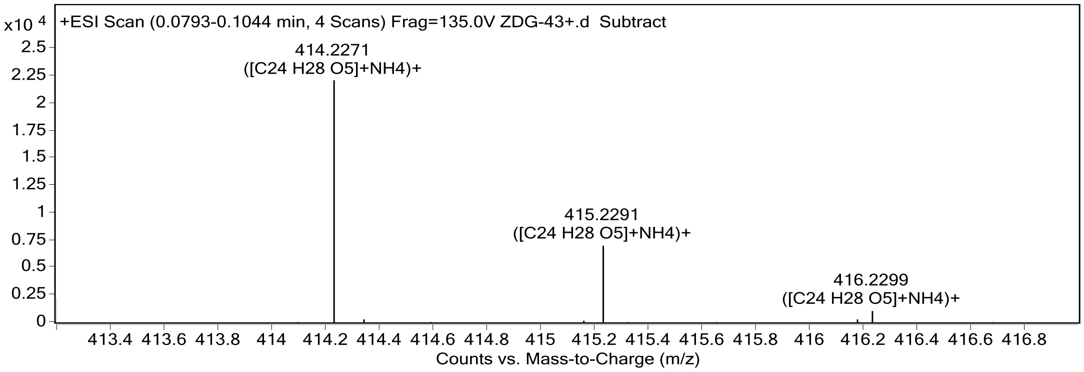

Peak List

m/z	z	Abund	Formula	Ion
189.0904	1	9958.61		
274.2736	1	4348.29		
318.2999	1	6436.74		
379.1902	1	30102.85		
380.1933	1	8048.27		
414.2271	1	22115.31	C24 H28 O5	(M+NH4)+
415.2291	1	7093.52	C24 H28 O5	(M+NH4)+
419.1824	1	10712.57		
810.4216	1	6444.87		
815.3765	1	5762.5		

Formula Calculator Element Limits

Element	Min	Max
C	3	80
H	0	100
O	0	20

Formula Calculator Results

Formula	CalculatedMass	CalculatedMz	Mz	Diff. (mDa)	Diff. (ppm)	DBE
C24 H28 O5	396.1937	414.2275	414.2271	0.40	0.97	11.0000

--- End Of Report ---

Figure S2.9 IR spectrum of compound 2

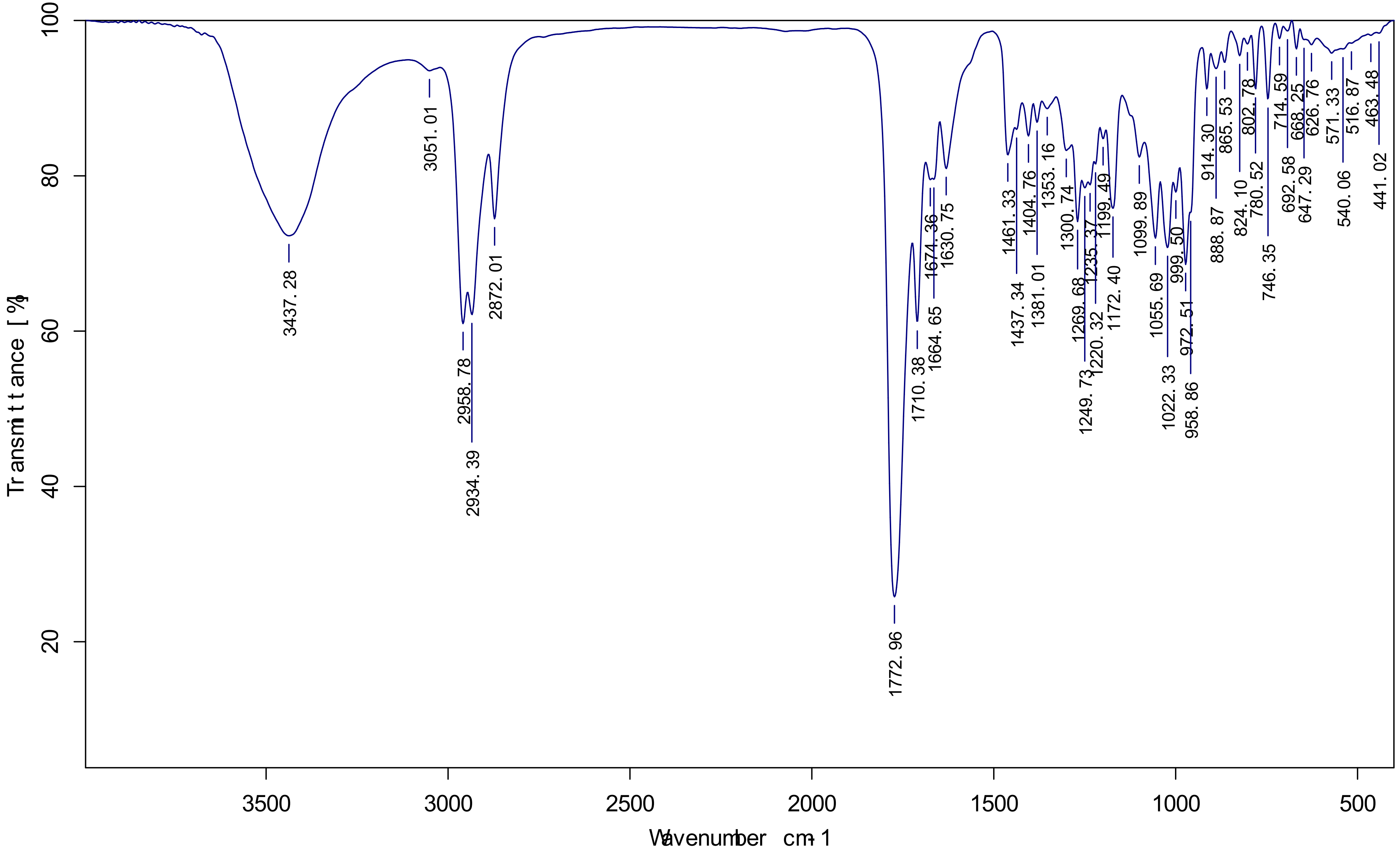

Sample Name: 2
Sample Form: KBr
Path of File: E:\data
Date of Measurement: 2024/10/8

Resolution: 4
Aperture Setting: 6 mm
Number of Background Scans: 16
Number of Sample Scans: 16

Beamsplitter Setting: KBr
Source Setting: MIR
Instrument Type: BRUKER VERTEX 70
Soft Version: OPUS8.1

Figure S2.10 ECD spectrum of compound (+)-2 in MeOH.
（Concentration: 0.1080 mg/mL MeOH）

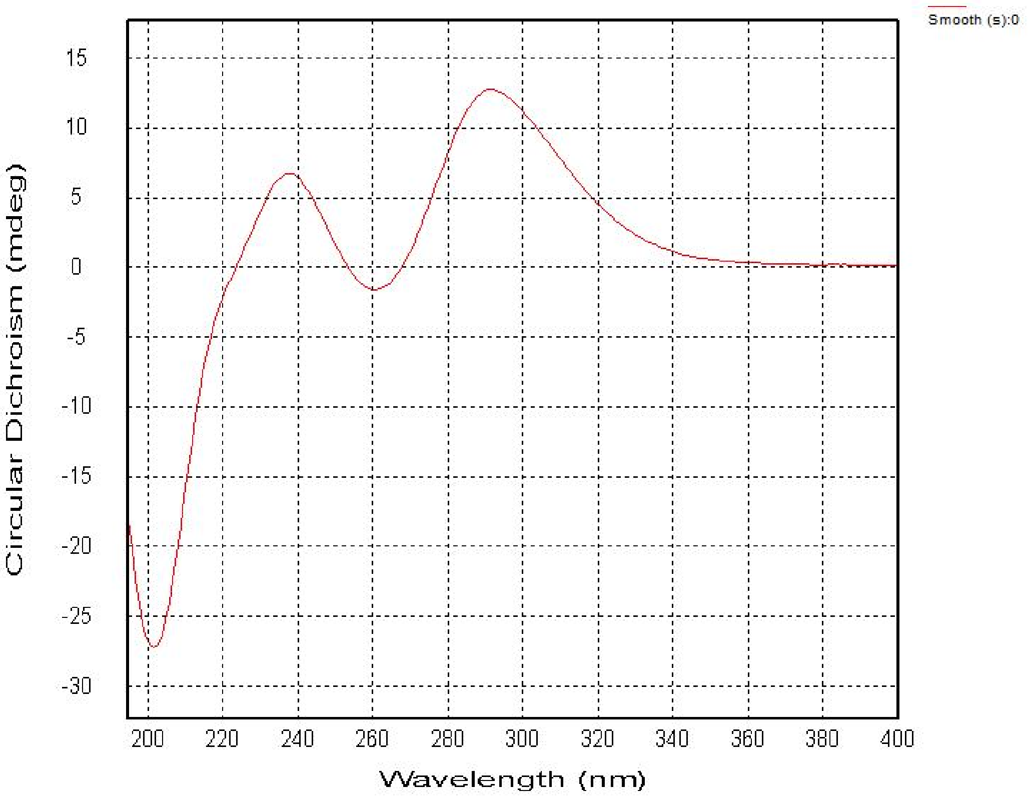

Figure S2.11 UV spectrum of compound (+) -2.

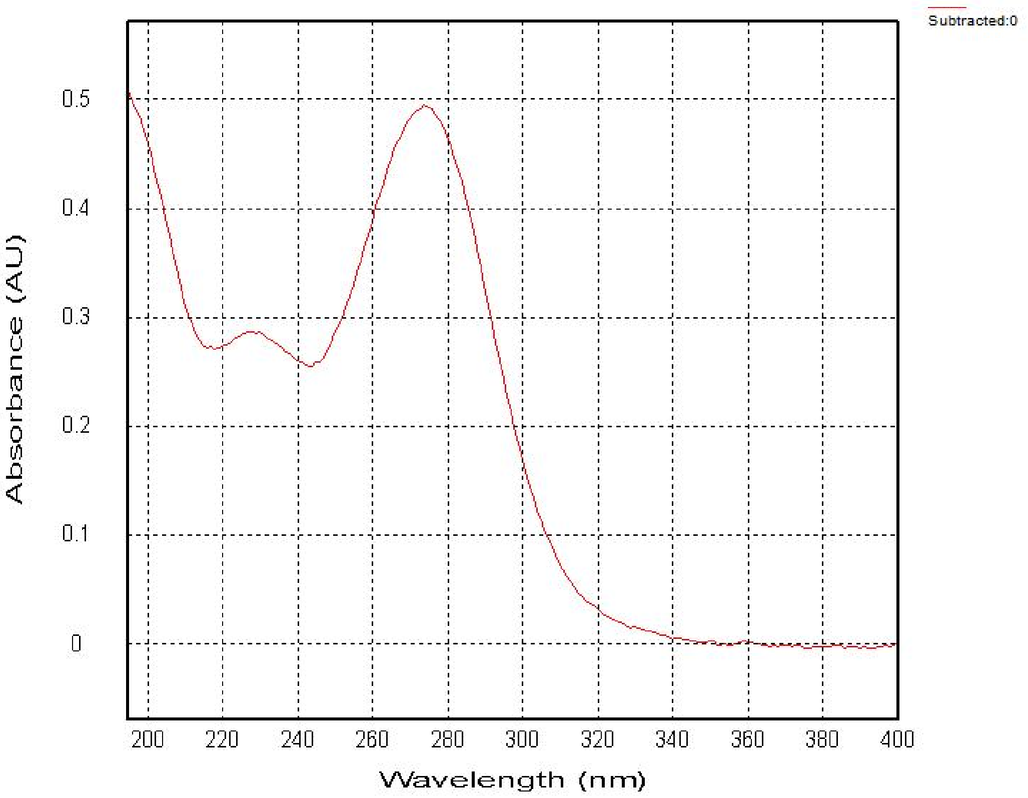

Figure S2.12 ECD spectrum of compound (-)-2 in MeOH.
（Concentration: 0.0650 mg/mL MeOH）

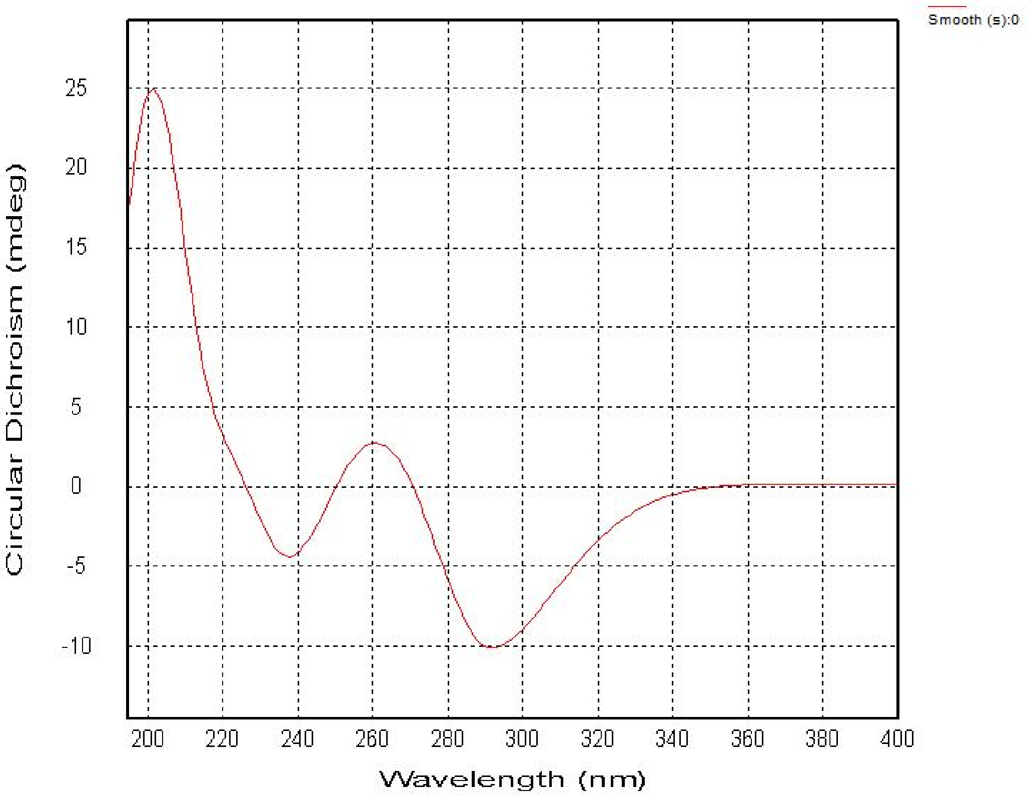

Figure S2.13 UV spectrum of compound (-) -2.

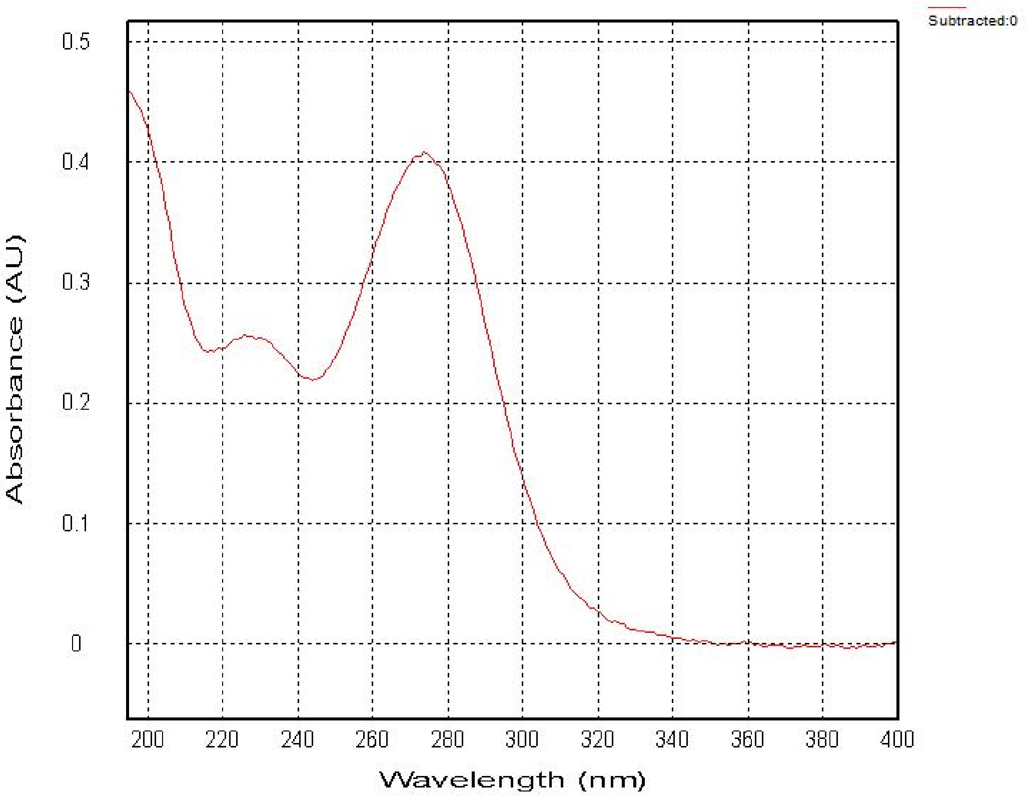

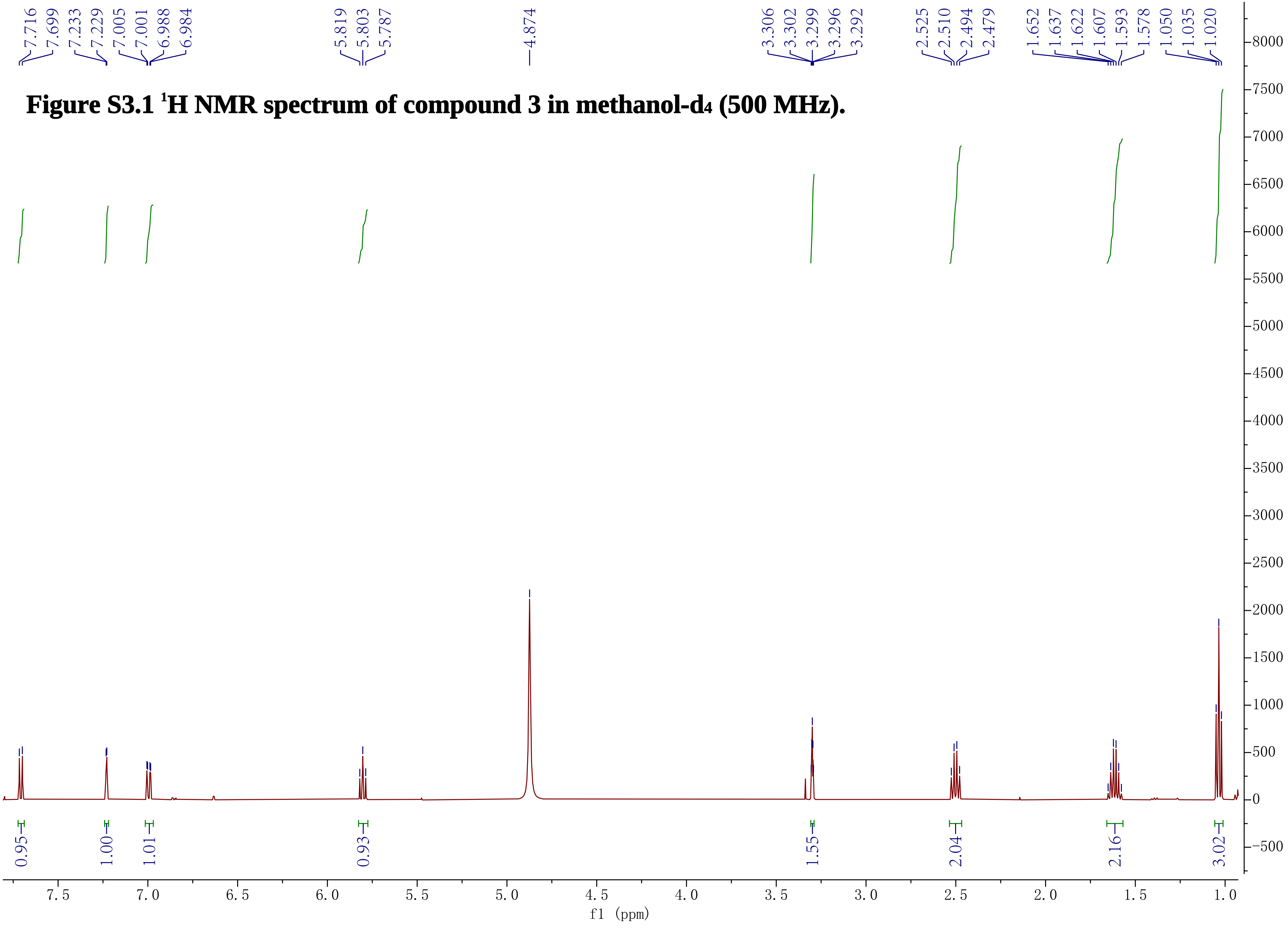

Figure S3.1 ^{1}H NMR spectrum of compound 3 in methanol-d_4 (500 MHz).

Figure S3.2 ^{13}C NMR spectrum of compound 3 in methanol-d_4 (125 MHz).

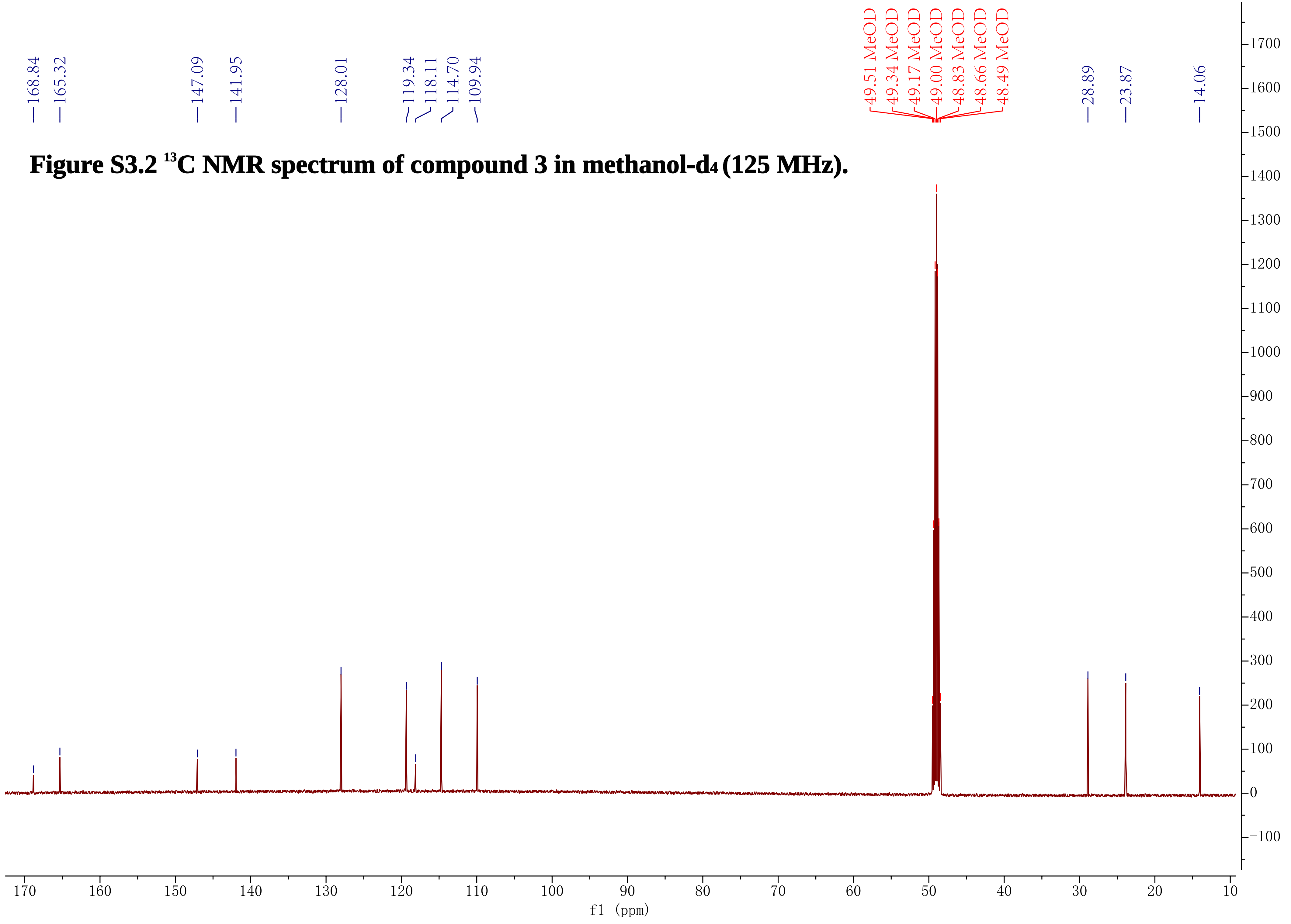

Figure S3.3 ^{1}H- ^{1}H COSY spectrum of compound 3 in methanol-d₄.

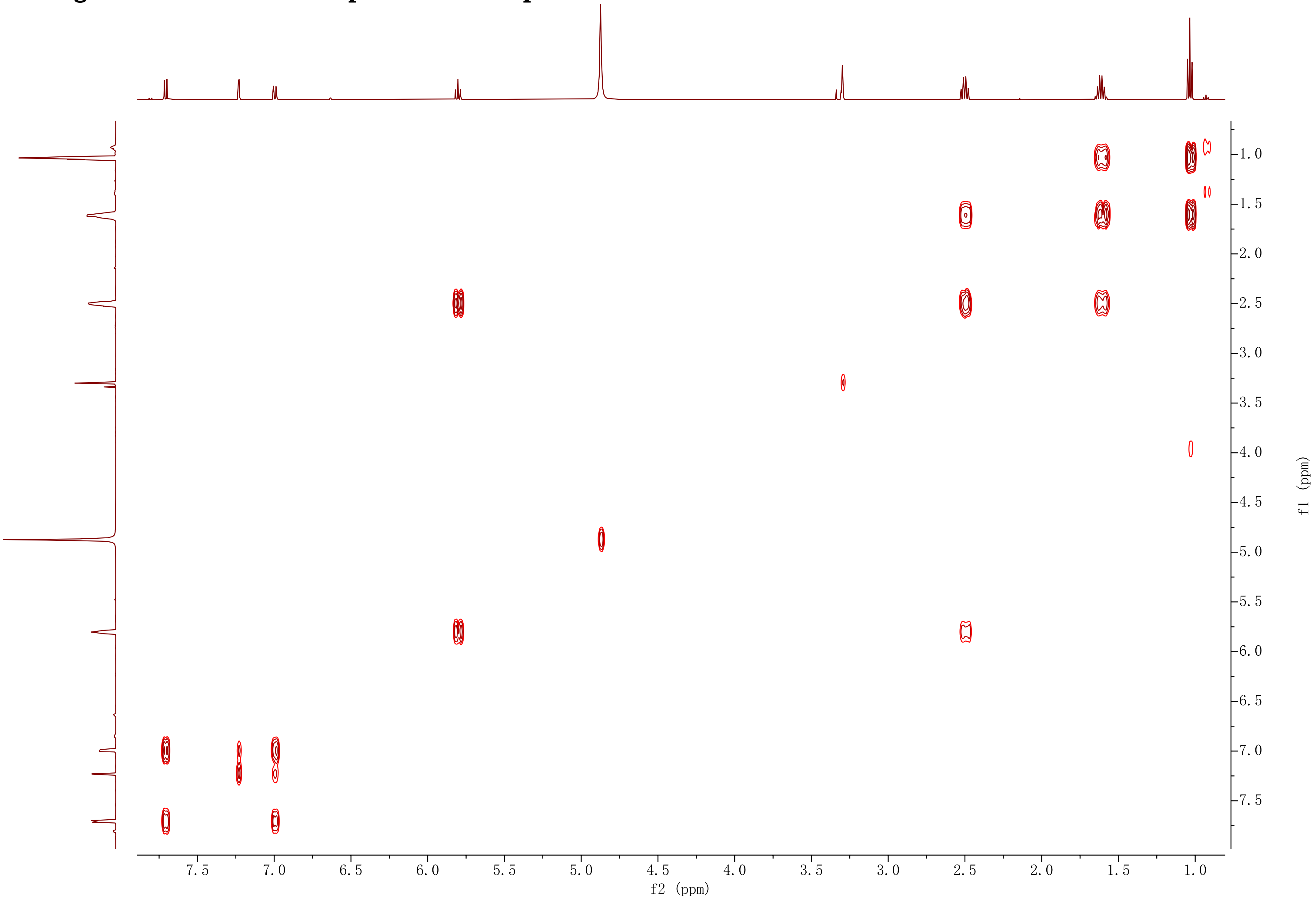

Figure S3.4 HSQC spectrum of compound 3 in methanol-d$_4$.

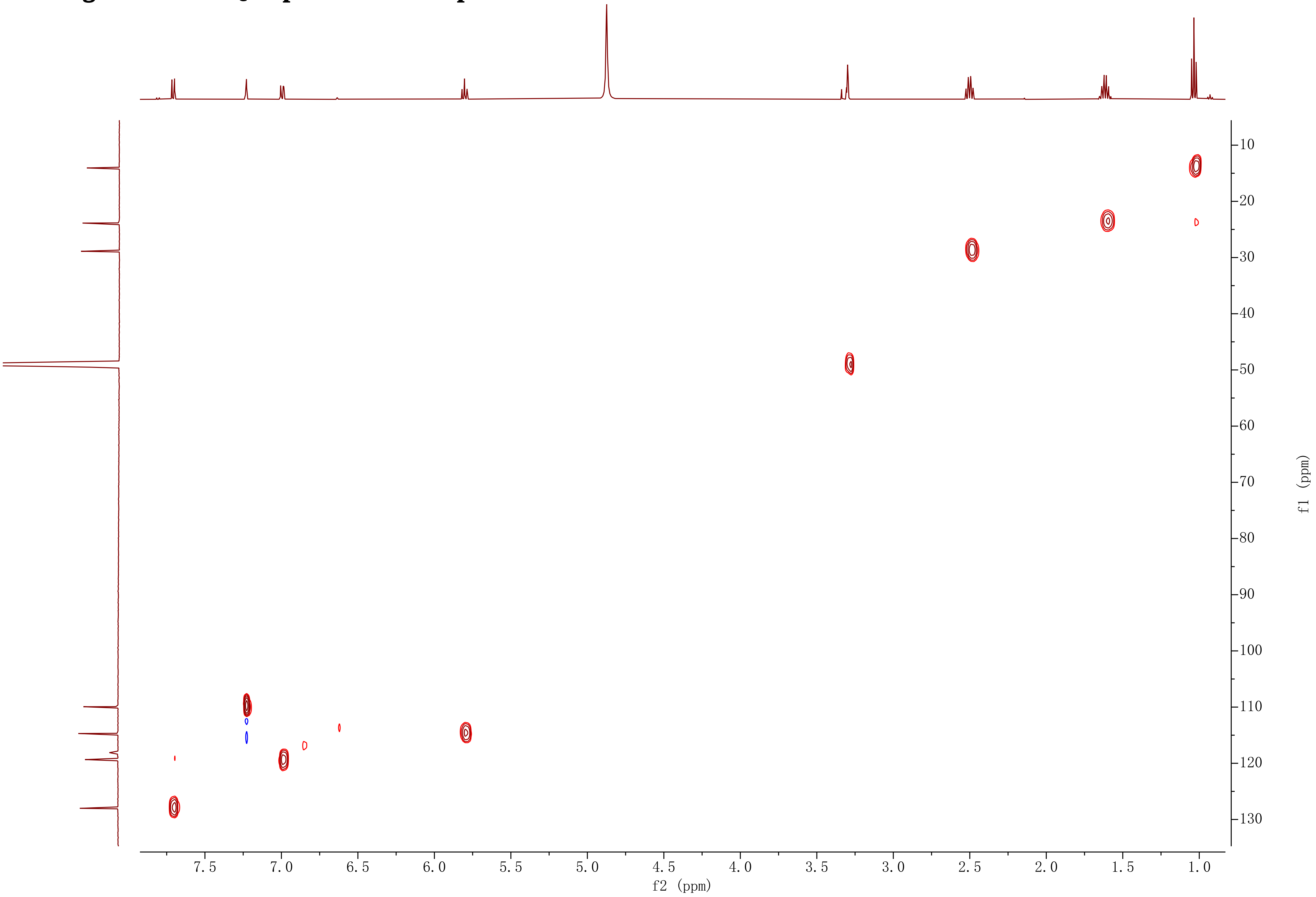

Figure S3.5 HMBC spectrum of compound 3 in methanol-d4.

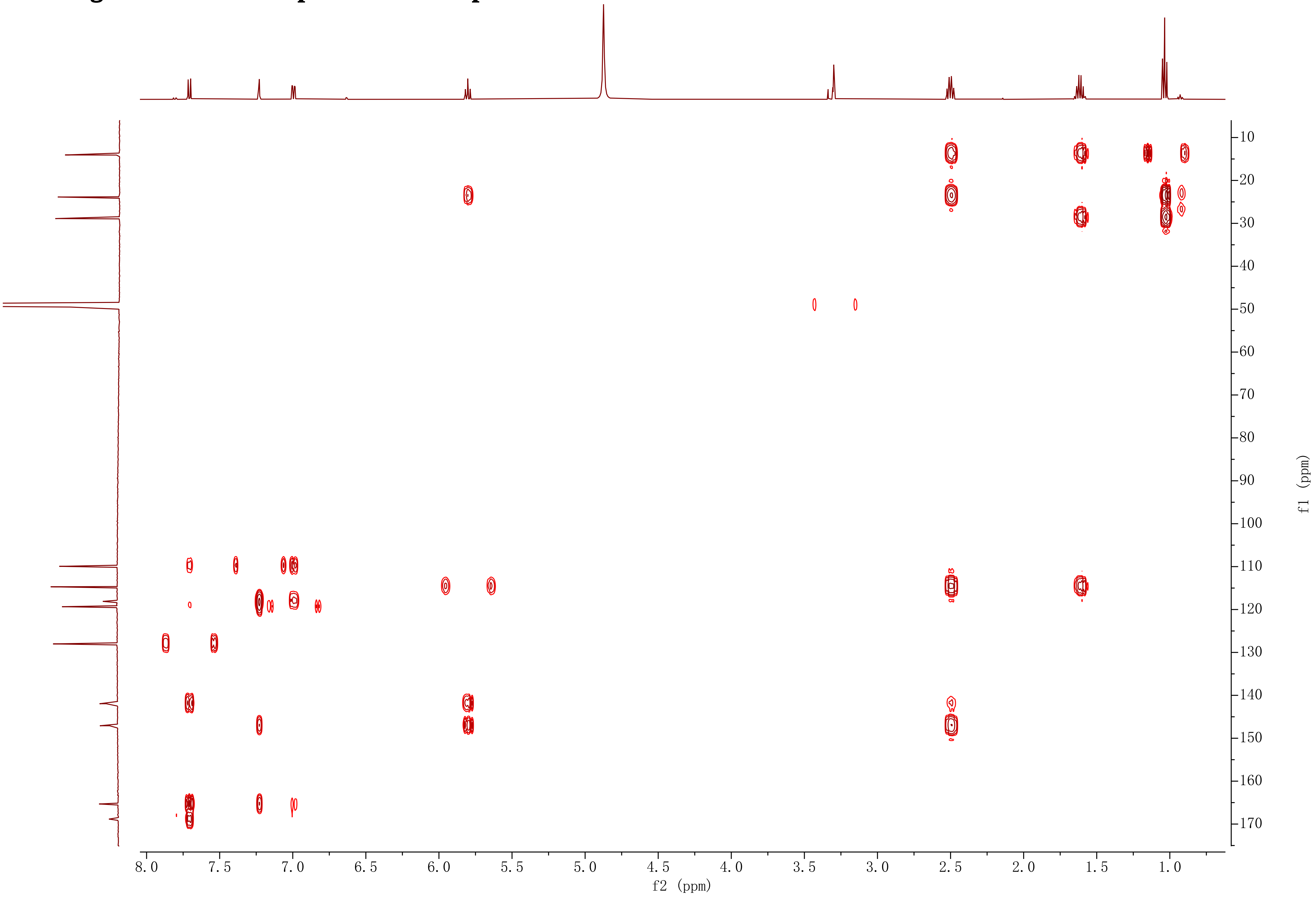

Figure S3.6 ROESY spectrum of compound 3 in methanol-d4.

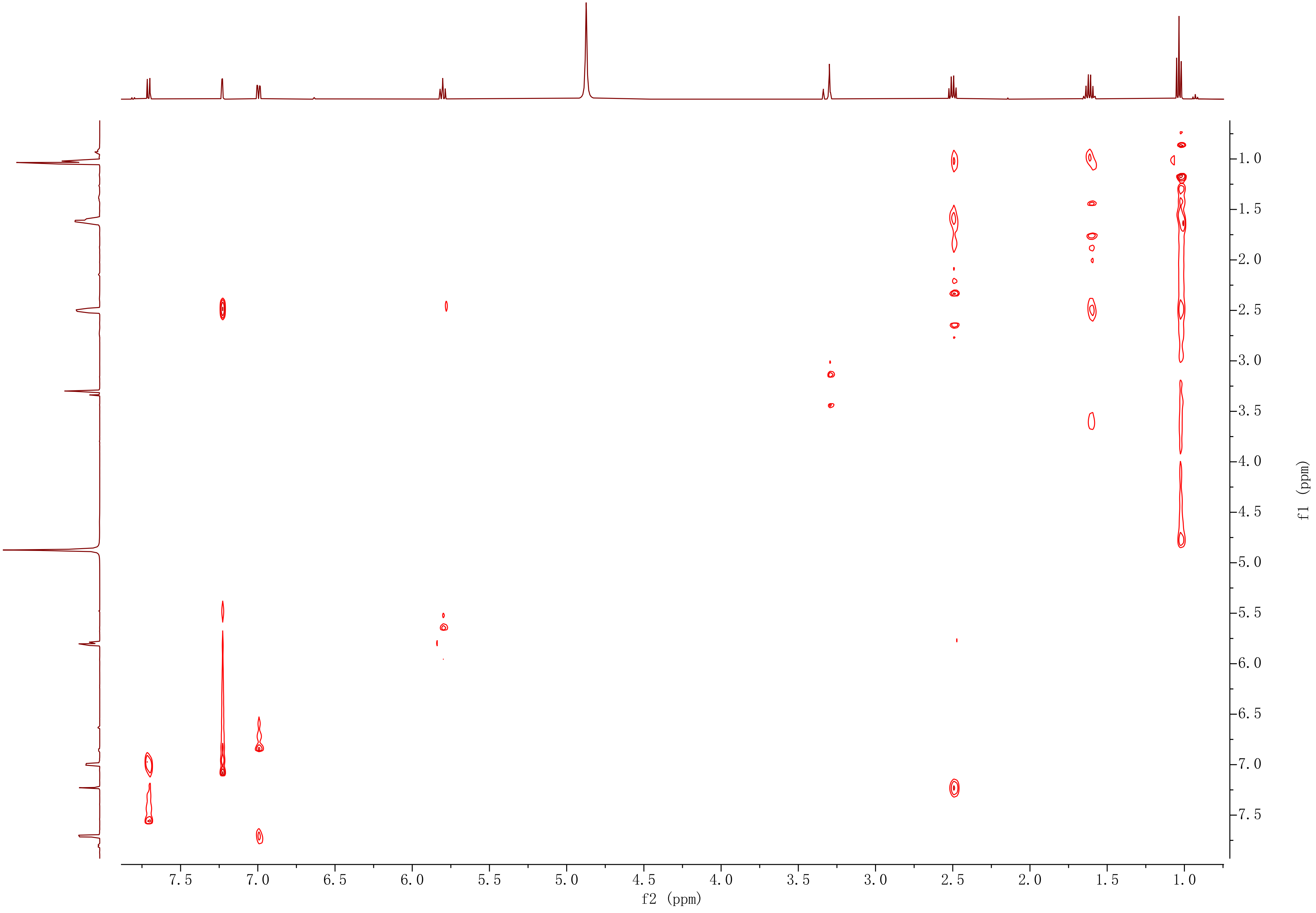

Figure S3.7 HR-ESI-MS spectrum of compound 3.

Qualitative Analysis Report

**Data Filename**	3.d	**Sample Name**	3
**Sample Type**	Sample	**Position**	P1-F4
**Instrument Name**	Instrument 1	**User Name**	
**Acq Method**	s.m	**Acquired Time**	1/21/2025 3:39:10 PM
**IRM Calibration Status**	Success	**DA Method**	PCDL.m
**Comment**			

Sample Group **Info.**
Acquisition SW Version 6200 series TOF/6500 series Q-TOF B.05.01 (B5125.2)

User Spectra

Fragmentor Voltage	Collision Energy	Ionization Mode
135	0	ESI

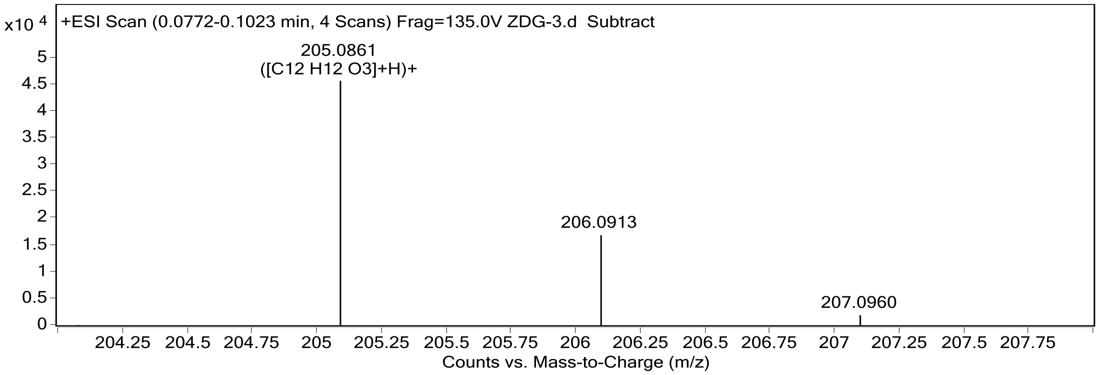

Peak List

m/z	z	Abund	Formula	Ion
187.0753	1	5455.15		
205.0861	1	45693.38	C12 H12 O3	(M+H)+
206.0913	1	17045.27		
227.0676	1	6367.14		
241.137	1	4896.23		
263.1192	1	8926.85		
264.1237	1	2165.99		
357.2032	1	4267.04		
431.1464	1	2631.59		
597.3332	1	2557.85		

Formula Calculator Element Limits

Element	Min	Max
C	3	70
H	0	200
O	1	30

Formula Calculator Results

Formula	CalculatedMass	CalculatedMz	Mz	Diff. (mDa)	Diff. (ppm)	DBE
C12 H12 O3	204.0786	205.0859	205.0861	-0.20	-0.98	7.0000

--- End Of Report ---

Figure S3.8 IR spectrum of compound 3

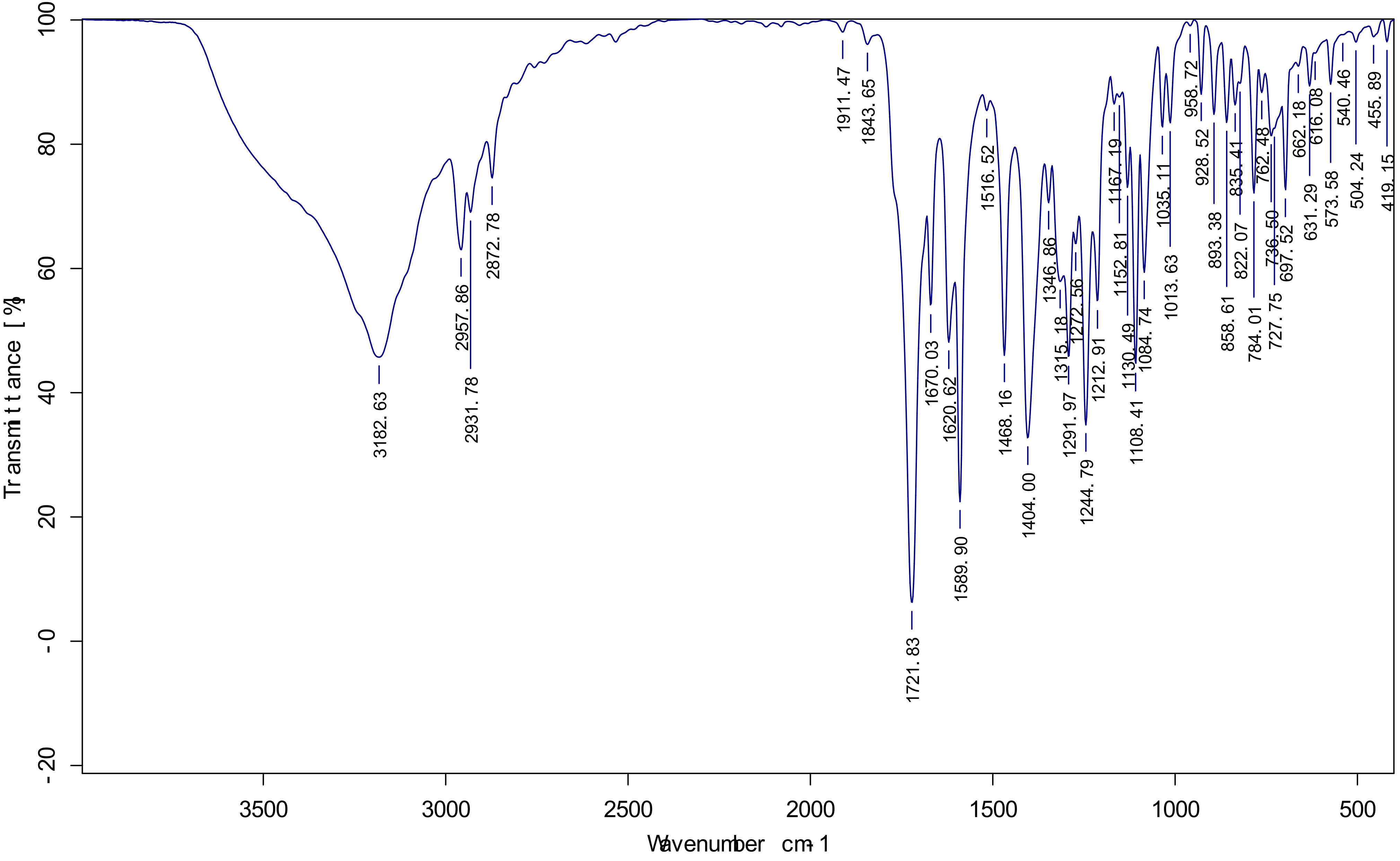

Sample Name: 3
Sample Form: KBr
Path of File: E:\data
Date of Measurement: 2025/1/23

Resolution: 4
Aperture Setting: 6 mm
Number of Background Scans: 16
Number of Sample Scans: 16

Beamsplitter Setting: KBr
Source Setting: MIR
Instrument Type: BRUKER VERTEX 70
Soft Version: OPUS8.1
